# Advancing the community health vulnerability index for wildland fire smoke exposure^☆^

**DOI:** 10.1016/j.scitotenv.2023.167834

**Published:** 2023-10-14

**Authors:** Jihoon Jung, Joseph L. Wilkins, Claire L. Schollaert, Yuta J. Masuda, John C. Flunker, Rachel E. Connolly, Savannah M. D’Evelyn, Eimy Bonillia, Ana G. Rappold, Ryan D. Haugo, Miriam E. Marlier, June T. Spector

**Affiliations:** a Department of City and Regional Planning, University of North Carolina, Chapel Hill, NC, USA; b Interdisciplinary Studies Department, Howard University, Washington, DC, USA; c School of Environmental and Forest Sciences, University of Washington, Seattle, WA, USA; d Department of Environmental & Occupational Health Sciences, University of Washington, Seattle, WA, USA; e Partnerships and Programs, Vulcan LLC, Seattle, WA, USA; f Department of Environmental Health Sciences, Jonathan and Karin Fielding School of Public Health, University of California, Los Angeles, CA, USA; g United States Environmental Protection Agency, Office of Research and Development, Durham, NC, USA; h The Nature Conservancy, Portland, OR, USA

**Keywords:** Vulnerability, Assessment, Wildland fire, PM_2.5_, Adaptive capacity, Sensitivity

## Abstract

Wildland fire smoke risks are not uniformly distributed across people and places, and the most vulnerable communities are often disproportionately impacted. This study develops a county level community health vulnerability index (CHVI) for the Contiguous United States (CONUS) using three major vulnerability components: adaptive capacity, sensitivity, and exposure at the national and regional level. We first calculated sensitivity and adaptive capacity sub-indices using nine sensitivity and twenty adaptive capacity variables. These sub-indices were then combined with an exposure sub-index, which is based on the Community Multiscale Air Quality data (2008–2018), to develop CHVI. Finally, we conducted several analyses with the derived indices to: 1) explore associations between the level of fine particulate matter from wildland fires (fire-PM_2.5_) and the sub-indices/CHVI; 2) measure the impact of fire-PM_2.5_ on the increase in the annual number of days with 12–35 μg/m^3^ (moderate) and >35 μg/m^3^ (at or above unhealthy for sensitive groups) based on the US EPA Air Quality Index categories, and 3) calculate population size in different deciles of the sub-indices/CHVI. This study has three main findings. First, we showed that the counties with higher daily fire-PM_2.5_ concentration tend to have lower adaptive capacity and higher sensitivity and vulnerability. Relatedly, the counties at high risk tended to experience a greater increase in the annual number of days with 12–35 μg/m^3^ and >35 μg/m^3^ than their counterparts. Second, we found that 16.1, 12.0, and 17.6 million people out of 332 million in CONUS reside in the counties in the lowest adaptive capacity decile, highest sensitivity decile, and highest vulnerability decile, respectively. Third, we identified that the US Northwest, California, and Southern regions tended to have higher vulnerability than others. Accurately identifying a community’s vulnerability to wildfire smoke can help individuals, researchers, and policymakers better understand, prepare for, and respond to future wildland fire events.

## Graphical Abstract

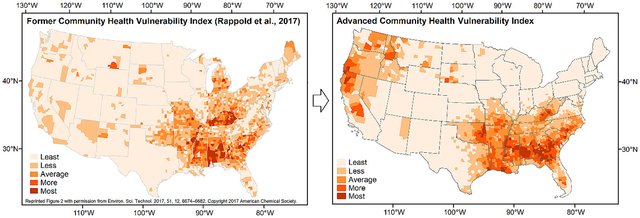

## 1. Introduction

Climate change has increased favorable conditions for wildfires (Goss et al., 2020; Jones et al., 2020), and humans have directly increased the risk of wildfires through decades of fire suppression efforts and exclusion of Indigenous cultural burning (Taylor et al., 2016), alteration of ecosystems and introduction of exotic species such as invasive annual grasses (Lambert et al., 2010), and rapid expansion of residential and recreational areas into the wildfire and wildland urban interface (Radeloff et al., 2018). The increased frequency of wildfires has led to a rise in wildfire smoke over the past few decades (Wilkins et al., 2018), with an estimated 27-fold increase in the number of people who have experienced at least one day of wildfire fine particulate matter (PM_2.5_) above 100 μg/m^3^ per year in the past decade (Childs et al., 2022). The substantial release of PM_2.5_ into the atmosphere has led to an increase in human mortality and morbidity. Roberts and Wooster (2021) estimated that the average annual global premature mortality attributable to landscape fire smoke exposure is 677,745, which corresponds to 0.52 % of total global mortality.

To date, a growing body of evidence suggests that the most vulnerable communities are often disproportionately impacted by wildland fire smoke (Rappold et al., 2017; Tessum et al., 2021, 2019), which exacerbates existing social and environmental injustices (e.g., Reid et al., 2016; Tessum et al., 2021, 2019). The impact of wildland fire smoke in a region is determined not only by the biophysical aspects of wildland fires such as frequency, intensity, and duration, but also by the demographic and socioeconomic factors in the region (Davies et al., 2018; Haikerwal et al., 2015; Ye et al., 2021). As a result, there is a need to identify where communities are most vulnerable and/or disproportionately impacted by wildland fire smoke, as well as transdisciplinary approaches to tackle such challenges (D’Evelyn et al., 2022).

We are aware of two efforts that have developed a vulnerability index to wildland fire smoke to identify vulnerable and/or disproportionately impacted communities. Rappold et al. (2017) introduced a county level community health vulnerability index (CHVI) based on wildland smoke simulated through the Community Multiscale Air Quality (CMAQ) model for the contiguous United States (CONUS) for the period between 2008 and 2012. This study identified that the counties along the western slope of the Appalachian Mountains have the highest vulnerability, with 10.3 million people experiencing >10 days/year with >35 μg/m^3^ PM_2.5_ as a result of wildland fires. They also found that the most vulnerable counties tend to experience more smoke exposures compared to less vulnerable communities. Another study done by Vaidyanathan et al. (2018) developed an online tool for identifying at-risk populations to wildfire smoke using historical locations of fires and burn severity, air quality, health data, and information on vulnerable populations.

Here, we build upon these wildland fire vulnerability studies in three respects. First, we incorporate adaptive capacity, the ability of a region to adapt to natural hazards to reduce potential damages, in the vulnerability assessment, which is crucial for accurately reflecting the actual vulnerability of a natural system, region, or community (Rappold et al., 2017; Smit and Pilifosova, 2003). Second, we conduct a regional assessment, which complements existing vulnerability assessments (e.g., Rappold et al., 2017) that conduct national level analyses. Third, we use a longer time series of wildfire smoke exposure data and update all variables in the study with recent datasets. The former study was based on only 5 years of data (e.g., Rappold et al., 2017), which did not capture the recent years of record wildfire activity in the Western US (https://www.nifc.gov). By addressing these gaps in previous research, the present study seeks to enhance the understanding of vulnerability to wildland fire smoke exposure. With these improvements, we address three questions in this study:

Q1: Which counties are more or less vulnerable to PM_2.5_ exposures solely from wildfires and prescribed fires (fire-PM_2.5_) at the national and regional level?

Q2: How are fire-PM_2.5_ exposures associated with the adaptive capacity and sensitivity sub-indices, as well as the overall vulnerability expressed by CHVI?

Q3: How many people reside in counties in the lowest adaptive capacity decile, highest sensitivity decile, and highest CHVI decile? Do the counties experience a greater increase in the annual number of days with 12–35 μg/m^3^ (moderate) or >35 μg/m^3^ (at or above unhealthy for sensitive groups) due to wildland fires than their counterparts?

## 2. Data and method

### 2.1. Vulnerability assessment

Vulnerability assessments are commonly used to measure the potential damage and life loss from hazardous events and disasters (Cutter, 1996). The assessments often refer to social vulnerability (Wang et al., 2022). Social vulnerability can be measured with the characteristics of a person or community that influence their capacity to anticipate, confront, repair, and recover from the adverse impacts of disasters (Cutter et al., 2012; Flanagan et al., 2011). Vulnerability assessments can help identify, compare, and quantify vulnerable geographic areas, subpopulations, and industrial sectors through the application of various analytical frameworks (Fuchs et al., 2012). Such frameworks can provide a tool for decreasing population vulnerability, increasing adaptive capacity, building resilience to cope with disasters, and preparing effective disaster prevention and reduction (Du et al., 2015). Because of their practicality, a number of studies have adapted this framework with different settings depending on the purpose of the studies to investigate vulnerability to multiple natural disasters such as heat waves (e.g., Reid et al., 2009), floods (e.g., Kaźmierczak and Cavan, 2011), hurricanes (e.g., Burton, 2010), and wildfires (e.g., Chuvieco et al., 2014; Davies et al., 2018).

Vulnerability assessments provide insight into a region’s vulnerability to hazards (Adger, 2006). In this study, we define vulnerability as a function of exposure, sensitivity, and adaptive capacity following existing literature (Füssel and Klein, 2006; IPCC, 2007; Yoo et al., 2014). Exposure is “the degree, duration, and extent in which a system (region) is in contact with, or subject to, the perturbation” (Kasperson et al., 2005), sensitivity is “the degree to which a system (region) is affected, either adversely or beneficially by natural hazards” (IPCC, 2014), and adaptive capacity is “the ability of a system (region) to adjust to natural hazards to moderate potential damages” (Gallopín, 2006). Functionally, vulnerability is inversely related to adaptive capacity and positively associated with exposure and sensitivity.

Based on the definitions and former literature (see Sections 2.2 through 2.4 below), we systematically collected a total of one exposure, nine sensitivity, and twenty adaptive capacity variables to estimate vulnerability at the national and regional level. For the aims of our analysis, we limited variables to those that come from secondary datasets, are nationally representative, have national coverage, and consistently contain adequate spatial (i.e., county) and temporal (i.e., annual average) resolutions. We derived variables from four sources: 1) US Census Bureau American Community Survey, 2) Centers for Disease Control and Prevention (CDC) National Environmental Public Health Tracking (https://www.cdc.gov/nceh/tracking/index.html), and 3) American Lung Association (https://www.lung.org/research/sota). More information on the choice of exposure, sensitivity, and adaptive capacity variables can be found below.

### 2.2. Exposure

Our main exposure variable is PM_2.5_ originating from wildland fires (fire-PM_2.5_) which cover both wildfire and prescribed fire. Using the same method of Wilkins et al. (2018), we estimated PM_2.5_ concentrations from 2008 to 2018 using the CMAQ modeling system with and without wildfires and prescribed fires. The difference between the two model products is the contribution of fire emissions (i.e., fire-PM_2.5_) to the ambient PM_2.5_ levels. Inputs to CMAQ included gridded meteorological fields, emissions data, and boundary conditions. For regional CMAQ model simulations, we used annual CONUS Weather Research and Forecasting model (WRF) simulations utilizing 12 km horizontal grid spacing and 35 vertical layers from the surface to 50 hPa at varying thickness for meteorological fields (Wilkins et al., 2022). The North American Mesoscale Model from the National Centers for Environmental Prediction provided initial and boundary conditions for WRF. We based input emissions on a 12 km CONUS domain with speciation for the Carbon-Bond 05/6r3 chemical mechanisms. We used version 5.0.1–5.3 of the CMAQ modeling system (Appel et al., 2021, 2017, 2013; Byun and Schere, 2006). In this study, we excluded the pandemic years as they represent highly skewed outliers for many air quality studies (e.g., Deng et al., 2022; Vichova et al., 2021). Details on the general model configuration can be found in Wilkins et al. (2018) and for the additional years summarized in Supplementary Material and Supplementary Table S1.

With the simulated fire-PM_2.5_ concentrations, we calculated the average of all grid cell values within each county at the daily level from 2008 to 2018. For this, we used the extract function in the raster package in R. This function returns the average values of the cells of a raster object that are covered by a polygon (i.e., county boundary), excluding cells that are only partly covered by the polygon. The daily values were then averaged to determine the daily average PM_2.5_ concentration for each county during the research period.

### 2.3. Sensitivity

We measured the county’s sensitivity based on the proportion of nine subpopulation groups that are highly sensitive to fire-PM_2.5_ within the county: 1) young population (<5 years), 2) elderly population (≥65 years), 3) agricultural and construction workers, 4) those with diabetes, 5) obesity, 6) hypertension, 7) adult asthma, 8) pediatric asthma, and 9) chronic obstructive pulmonary disease (COPD). Previous studies have demonstrated that there is a greater adverse impact of smoke on those under 5 years (Ye et al., 2021) due to their smaller airways and higher ventilation rate compared with adults (Jacobson et al., 2012), and over 65 years (Haikerwal et al., 2015) due to the gradual decline in physiological processes over time and a higher prevalence of preexisting conditions among them (Sacks et al., 2011). We also included the proportion of workers (agricultural and construction workers) who are more likely to be exposed to PM_2.5_ through outdoor work and an increased respiratory rate associated with physical labor (Liu et al., 2021). Furthermore, we considered those having pre-existing health problems which are associated with higher risk of adverse health outcome following PM_2.5_ exposures, such as diabetes (Bateson and Schwartz, 2004; Mahsin et al., 2022), obesity (Siregar et al., 2022), hypertension (Reid et al., 2016), asthma (Stowell et al., 2019), and COPD (Bateson and Schwartz, 2004). Additional details (e.g., unit, source, data year) and the explanatory summary (e.g., mean, standard deviation, maximum, minimum) of these variables can be found in Supplementary Tables S2 and S3 respectively.

### 2.4. Adaptive capacity

We capture adaptive capacity by examining variables associated with a county’s ability to either mitigate or exacerbate the impacts of fire-PM_2.5_ exposures on population vulnerability. For this, we selected twenty variables consisting of six demographic, nine socioeconomic, and five infrastructure variables derived from the literature and constrained by data availability.

For demographic variables, we included five different demographic subpopulation groups which are commonly considered as vulnerable subpopulation groups during wildfire events: 1) disabled, 2) Black, 3) Hispanic, 4) American Indian and Alaska Native, 5) single parent households (Davies et al., 2018; Hwang and Meier, 2022). These groups are relatively more likely to have a limited adaptive capacity possibly due to their physical (Gaskin et al., 2017) or financial constraints (Cox and Kim, 2018). Hispanics and African Americans may be more likely to have higher reluctance toward fire mitigation practices due to their cultural, historical, or political experiences (Bowker et al., 2008; Davies et al., 2018; Nagler, 2017), which could in turn reduce their adaptive capacity. In addition to the five vulnerable subpopulation groups, we included the percentage of the population in the workforce to determine how many people would be available to participate in response to wildfire events, such as assisting with community evacuations (Smith, 2016).

For socioeconomic variables, we chose nine socioeconomic subpopulation characteristics which are highly associated with vulnerability to fire-PM_2.5_: 1) income, 2) poverty, 3) unemployment rate, 4) English proficiency, 5) education, 6) mobile home, 7) multi-housing unit, 8) households without car, and 9) population without health insurance. Communities characterized by low income, high poverty, and high unemployment rates have comparatively limited financial capacity to cover the costs of fire mitigation services (e.g., tree thinning), fire insurance, rebuilding, and community firefighting resources required to extinguish fires (Collins and Bolin, 2009; Davies et al., 2018; Mercer and Prestemon, 2005), which reduces a community’s adaptive capacity. We also include English proficiency and education (high school diploma), as those with limited English proficiency and low education attainment are more likely to have difficulties in accessing relevant information, recovering from disasters, and communicating/networking with others (Flanagan et al., 2011; Fothergill and Peek, 2004; Muttarak and Lutz, 2014). In addition, we added housing types (mobile home, multi-unit house) and vehicle ownership. Better housing quality and transportation access could provide more smoke-resistant indoor environments and reliable transportation to evacuate from high smoke areas (Brodie et al., 2006; Fatemi et al., 2017). Furthermore, we included the percentage of population with health insurance, which is assumed to be associated with adaptive capacity of people for mitigating the negative health impacts of fire-PM_2.5_ (Vo and Van, 2019).

Finally, we selected five infrastructure variables which could be used to effectively respond to a hazard by providing evacuation routes (road density), supporting health care services (number of hospitals, Pharmacies, and drug stores, healthcare support occupations), and supplying protective services (firefighters and other protective service workers). These could hypothetically be used by those communities exposed to fire-PM_2.5_. Additional details (e.g., unit, source, data year) and the explanatory summary (e.g., mean, standard deviation, maximum, minimum) of the variables can be found in Supplementary Tables S4 and S5 respectively.

### 2.5. Analytic methods

Our analytic framework is divided into two parts: 1) developing a CHVI and 2) evaluating the calculated adaptive capacity and sensitivity sub-indices, as well as CHVI, alongside fire-PM_2.5_ exposures. To develop the CHVI, we employed a deductive method. Unlike the inductive method, which is based on statistical relationships, the deductive method is built on prior theory and knowledge from previous studies without any additional rationale for the selection of those variables (Yoon, 2012). Because of the method’s flexibility, each study has a different set of variables for assessing the degree of vulnerability depending on research purpose and subject (e.g., Mucke, 2012; Shepard et al., 2012). Compared to inductive methods based on statistical methods (e.g., principal component analysis), our approach better helps communities by directly identifying what components (exposure, sensitivity, adaptive capacity) are elevating or lowering the degree of vulnerability.

Our study generated a vulnerability index specifically for wildland fire smoke. We achieved this by selecting variables that have been previously reported to be highly associated with wildland fire smoke and fire-related health outcomes, instead of using a pre-existing index such as the CDC/ATSDR Social Vulnerability Index (SVI) which focuses on broader and general emergency events (https://www.atsdr.cdc.gov/placeandhealth/svi/index.html). After collecting a total of one exposure, nine sensitivity, and twenty adaptive capacity variables, we assigned each variable’s direction based on the relationship between vulnerability and the variable. If a unit increase (e.g., percent) of the variable (e.g., proportion of households below poverty) elevates the degree of vulnerability, we assign plus (+) as its direction. On the other hand, if a unit increase (e.g., percent) of the variable (e.g., proportion of population in workforce) lowers the degree of vulnerability, we assign minus (−) as its direction. Each variable is then standardized to a scale between 0 and 1 because each variable has its own unit and direction (Eq. 1). Here, a score of 0 means the lowest exposure, the lowest sensitivity, and the highest adaptive capacity, and a score of 1 represents the highest exposure, the highest sensitivity, and the lowest adaptive capacity. Note that a low value on the “adaptive capacity sub-index” actually indicates a high level of adaptive capacity, whereas a high value on the “adaptive capacity sub-index” signifies a low level of adaptive capacity. After standardization, if the direction of variables is minus (e.g., proportion of population in workforce), we subtracted the standardized values from 1 to match the adaptive capacity scale with the highest adaptive capacity being 0 and the lowest adaptive capacity being 1.

(1) $$V_{ij}=\frac{\left(X_{ij}-MinX_{i}\right)}{\left(MaxX_{i}-MinX_{i}\right)}$$

where V_*ij*_ is the standardized value associated with the *i*th county for variable *j*; X_*ij*_ is the initial value of the *i*th county for variable *j*; MaxX_*i*_ and MinX_*i*_ represent the maximum and minimum value of variable *j*.

Next, the three sub-indices were separately calculated for exposure, sensitivity, and adaptive capacity by averaging all variables within each component (i.e., exposure, sensitivity, adaptive capacity). In this process, we weighted each variable equally. Unequal weight could be derived from statistical (e.g., factor analysis, regression coefficients) or participatory approaches (e.g., focus group discussions, surveys), but in practice neither approach has achieved primacy when determining weights, and unequal and subjective weighting could bring more uncertainty and errors than the equal weighting (Tate, 2013). For these reasons, many studies have applied equal weights in index calculations (e.g., Aubrecht and Özceylan, 2013; Chow et al., 2012; Vescovi et al., 2005).

Finally, the exposure, sensitivity, and adaptive capacity sub-indices were multiplied to generate a composite overall CHVI after being standardized to a scale between 0 and 1 with the same weight (Eq. (2)). Here, we used a multiplicative method instead of an additive method because it integrates the interplay of the three components in the vulnerability index. For example, if a county has no fire-PM_2.5_ exposure, that county should have the least vulnerability, as there is no environmental exposure and risk. However, with the additive method, the counties with high sensitivity and low adaptive capacity could have high vulnerability regardless of exposure levels, as the vulnerability is the sum of sensitivity, adaptive capacity, and exposure indices. We also conducted the same analysis with the additive method as a sensitivity analysis.


(2) Vulnerability=Exposure×Sensitivity×Adaptive capacity

**Fig. 1.**
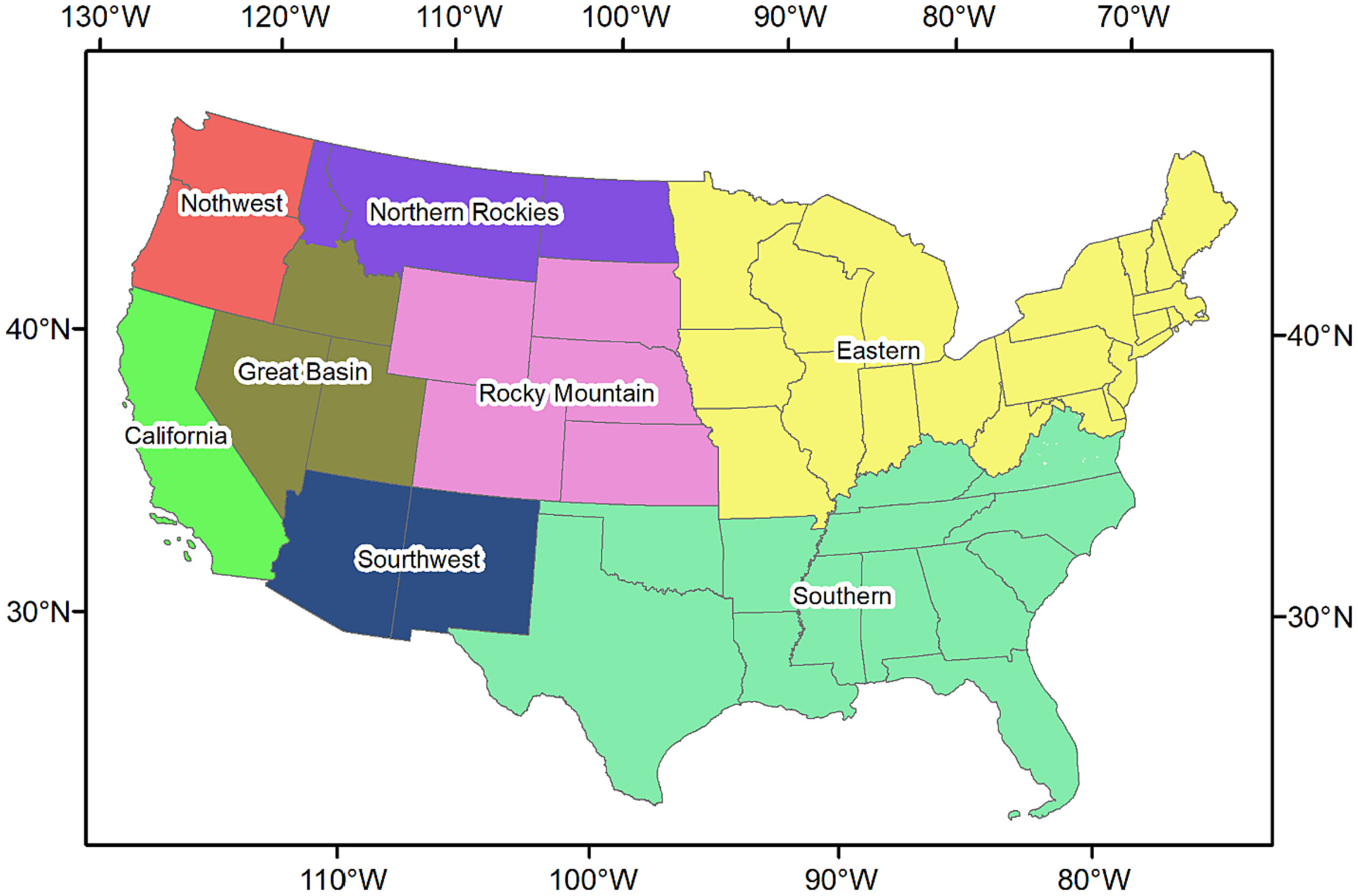
The interagency geographic areas for wildland fire management made by the National Interagency Fire Center.

We repeated the same procedures to calculate regional level CHVI which is based on the interagency geographic areas for wildland fire management made by National Interagency Fire Center (https://gacc.nifc.gov/) (Fig. 1). With these designations, the US is divided into 9 distinct geographic areas with the purpose of effective incident management and mobilization of resources such as people, aircraft, and ground equipment. For simplicity, we merged the Northern California region with the Southern California region in this paper. The calculated CHVI can aid in the development of region-specific strategies and the implementation of measures to mitigate vulnerability effectively. All processes for developing the overall CHVI are summarized in Supplementary Fig. S1.

Note that the method adopted in this paper does not need to consider multicollinearity among the variables. Multicollinearity is often problematic when statistical regression models are used. In this case, multicollinearity would flip the sign of regression coefficients or inflated the coefficients, which weakens the statistical power of the regression model. Our method, however, does not use statistical regression models. Instead, we employ the average of standardized variables following the method the CDC/ATSDR SVI adopted. The advantage of this method is that the presence of strong correlations among variables could potentially lead to an implicit weighting within an equal weighting system (Tate, 2013).

For the second part of the analysis, we first examined the association of the level of fire-PM_2.5_ exposure with the adaptive capacity sub-index, sensitivity sub-index, and CHVI using Spearman correlation coefficients. In this process, we also tabulated the statistical summary (i.e., mean, max, min, and standard deviation) of daily fire-PM_2.5_ and the number of counties corresponding to certain daily fire-PM_2.5_ levels (i.e., 0.00–0.15, 0.15–0.75, 0.75–1.50, >1.50 μg/m^3^) by decile for each index. We then investigated the inequality in the derived sub- and overall indices with the Gini coefficients (Gini, 1912) to understand which index is distributed more evenly or less evenly. Gini coefficients range from zero to one, with zero referring to perfect equality in the sub- and overall indices, which means the index values of all counties are the same. On the other hand, one indicates perfect inequality in the indices.

Next, we calculated the impact of fire-PM_2.5_ on the increase in the annual number of days with 12–35 μg/m^3^ (moderate) and >35 μg/m^3^ (at or above unhealthy for sensitive groups), following the US Environmental Protection Agency (EPA) Air Quality Index (AQI) categories, categorized by the deciles of the sub-indices and CHVI. For this, we separately counted the annual number of days between 12 and 35 μg/m^3^ (moderate air quality days) and >35 μg/m^3^ (at or above unhealthy air quality days for sensitive groups) for both all-sources PM_2.5_ and all-sources PM_2.5_ excluding wildland fires. The difference between the two model products lies in the extent to which fire-PM_2.5_ contributes to the increase in the number of moderate and at or above unhealthy air quality days/year for sensitive groups. Finally, we calculated population size at risk, categorized by fire-PM_2.5_ levels, adaptive capacity sub-index, sensitivity sub-index, and CHVI’s decile.

## 3. Results

### 3.1. Spatial patterns of the calculated indices

**Fig. 2.**
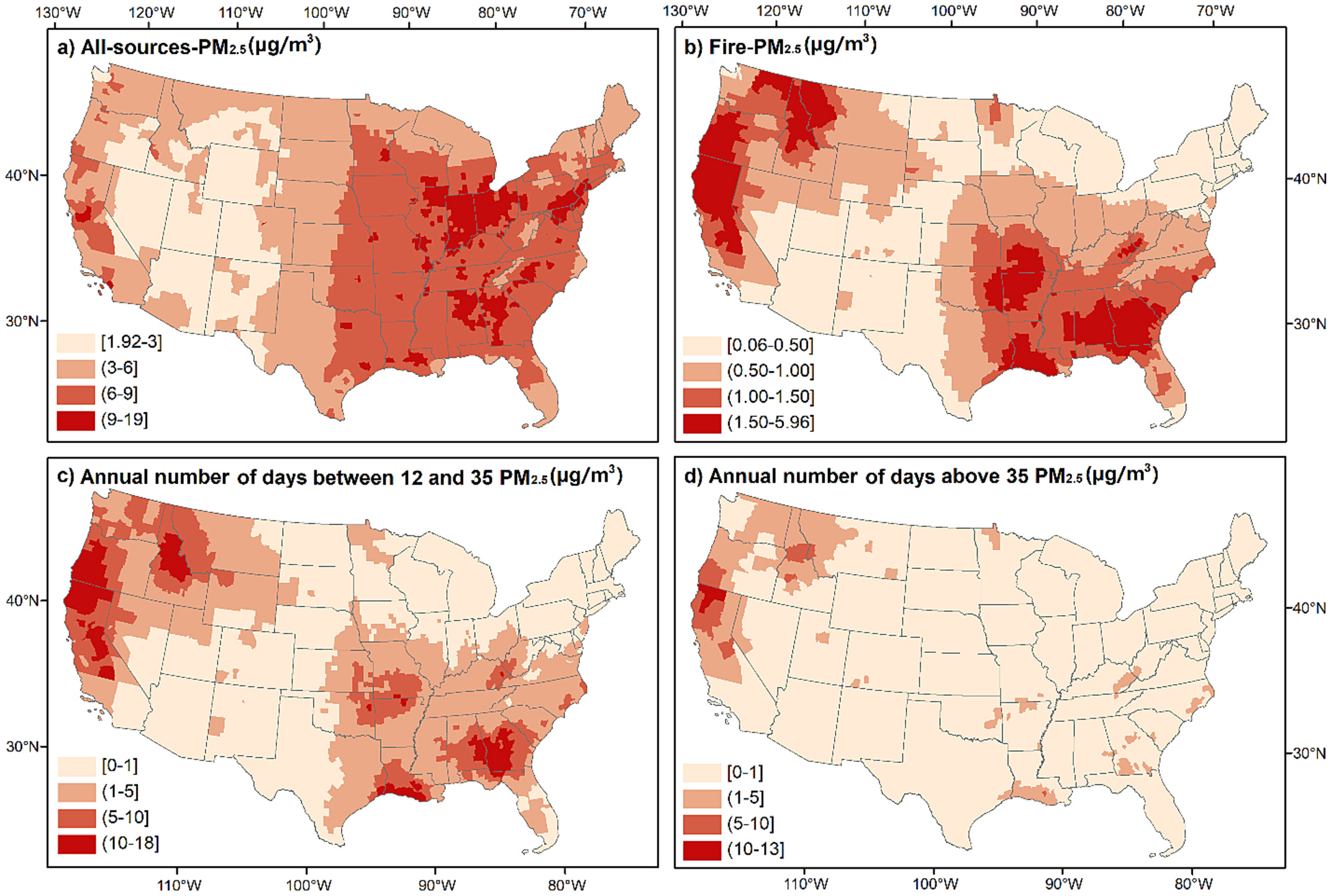
(a) Daily averaged all-sources PM_2.5_ (μg/m^3^) by county from 2008 to 2018, (b) Daily averaged fire-PM_2.5_ (μg/m^3^) by county from 2008 and 2018, (c) Annual number of days between 12 and 35 fire-PM_2.5_ (μg/m^3^), (d) Annual number of days above 35 fire-PM_2.5_ (μg/m^3^).

We estimated daily PM_2.5_ exposure from 2008 to 2018 using the CMAQ model with (i.e., all-sources-PM_2.5_) and without (i.e., fire-PM_2.5_) wildfires and prescribed fires. The difference between the two model products is the contribution of wildfires and prescribed fires to the ambient PM_2.5_ levels (i.e., fire-PM_2.5_). All-sources-PM_2.5_ (Fig. 2a) and fire-PM_2.5_ (Fig. 2b) showed different spatial patterns. All-sources-PM_2.5_ exhibited higher PM_2.5_ concentrations in the Eastern (7.90 μg/m^3^) and Southern (7.49 μg/m^3^) regions, while we observed higher fire-PM_2.5_ concentrations in the California (1.97 μg/m^3^), Northwest (1.35 μg/m^3^), and Southern (1.16 μg/m^3^) regions. Fig. 2c and d respectively represent the annual number of days between 12 and 35 μg/m^3^ (moderate air quality days) and >35 μg/m^3^ (at or above unhealthy air quality days for sensitive groups) due to wildland fire PM_2.5_. The annual number of days between 12 and 35 μg/m^3^ (Fig. 2c) tend to show a similar spatial pattern with fire-PM_2.5_ concentrations (Fig. 2b). On the other hand, we observed a distinct spatial pattern in the annual number of days with fire-PM_2.5_ >35 μg/m^3^ (Fig. 2d). Most counties having a high number of days >35 μg/m^3^ were concentrated in the California (3.62 days/year) and Northwest (1.88 days/year) regions. More details on the average concentration levels and the number of days between 12 and 35 μg/m^3^ and >35 μg/m^3^ by region can be found in Supplementary Table S6.

**Fig. 3.**
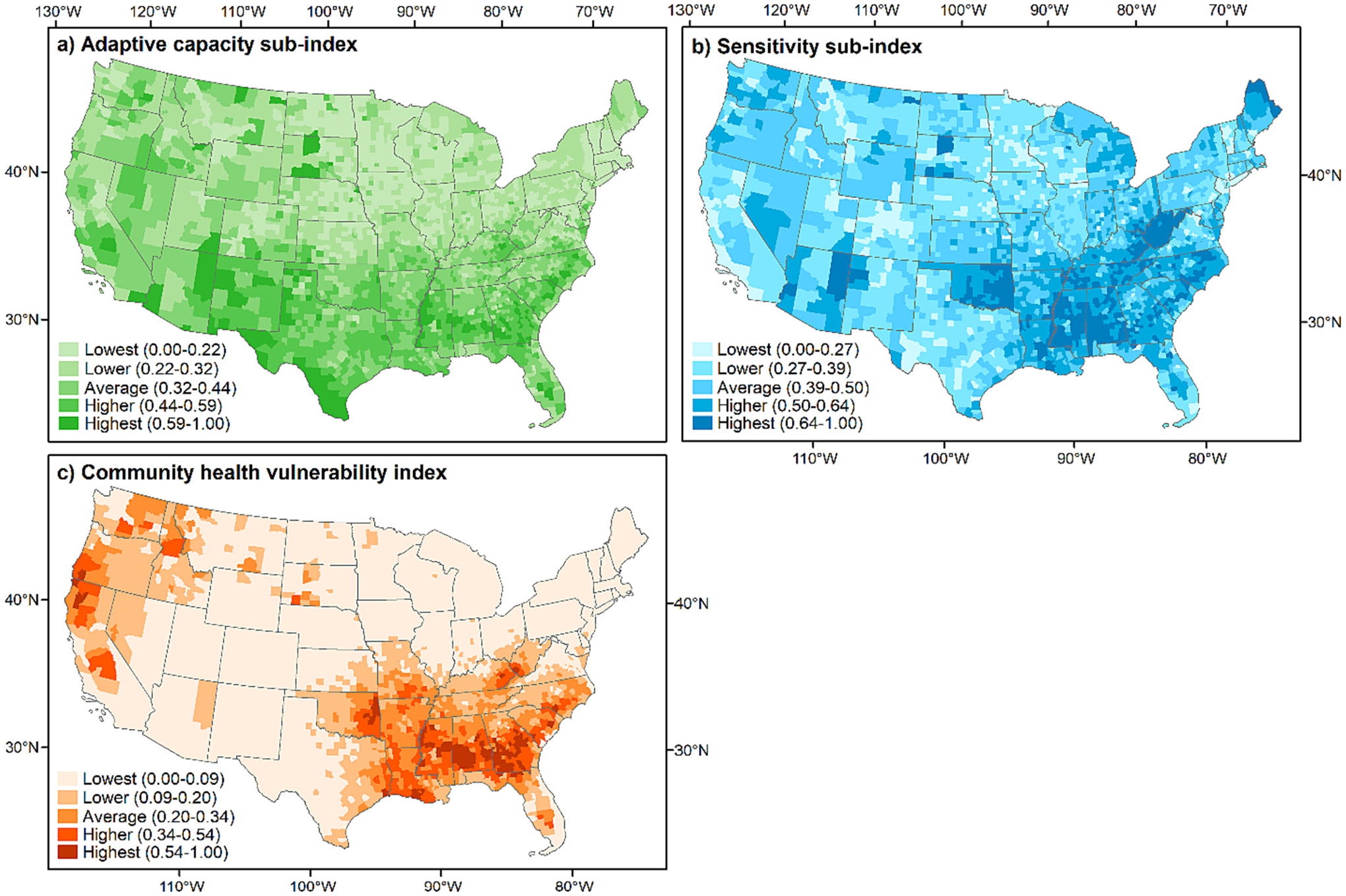
Sub- and overall indices classified into five categories using the natural Jenks method.

We separately calculated adaptive capacity (Fig. 3a) and sensitivity (Fig. 3b) sub-indices. Note that a low value on the adaptive capacity sub-index implies a strong adaptive capacity, while a high value indicates a weak adaptive capacity. Overall, we observed higher adaptive capacity sub-index values in the Southwest (0.49) and Southern (0.43) regions compared to the Eastern (0.25), Northern Rockies (0.26), and Rocky Mountain (0.28) regions. The spatial pattern of the sensitivity sub-index differed from the adaptive capacity sub-index. The Southern (0.52) region tended to have higher sensitivity sub-index values than other regions. We especially observed higher sensitivity in the states of Mississippi, Louisiana, Alabama, Oklahoma, and West Virginia. For CHVI, we found two clusters (Fig. 3c). One is in the Southern (0.24) region covering Georgia, Alabama, Mississippi, Louisiana, and Oklahoma. The other is in the Northwest (0.16) and California (0.18) regions covering Washington, Oregon, California, and Idaho. Readers can find more information on descriptive summaries of sensitivity and adaptive capacity sub-indices and CHVI by region in Supplementary Table S7.

As a sensitivity analysis, we also tested the additive method to examine if there is a large difference between the multiplicative method and additive method. The result shows that there were no large differences in high vulnerability areas we are interested in within this paper (Supplementary Figs. S2 and S3). However, there were significant differences in low vulnerability areas. The differences in low vulnerability areas may result from the different methods we used. The multiplicative method represents areas with no exposure as zero, while the additive method represents the same areas with the sum of the adaptive capacity sub-index and sensitivity sub-index. For more information, we also compared the top one hundred most and least vulnerable counties using both multiplicative and additive methods, respectively. Results show that 75 out of the top 100 most vulnerable counties and 58 out of the top 100 least vulnerable counties were consistently identified by both methods (Supplementary Tables S8–S11).

**Fig. 4.**
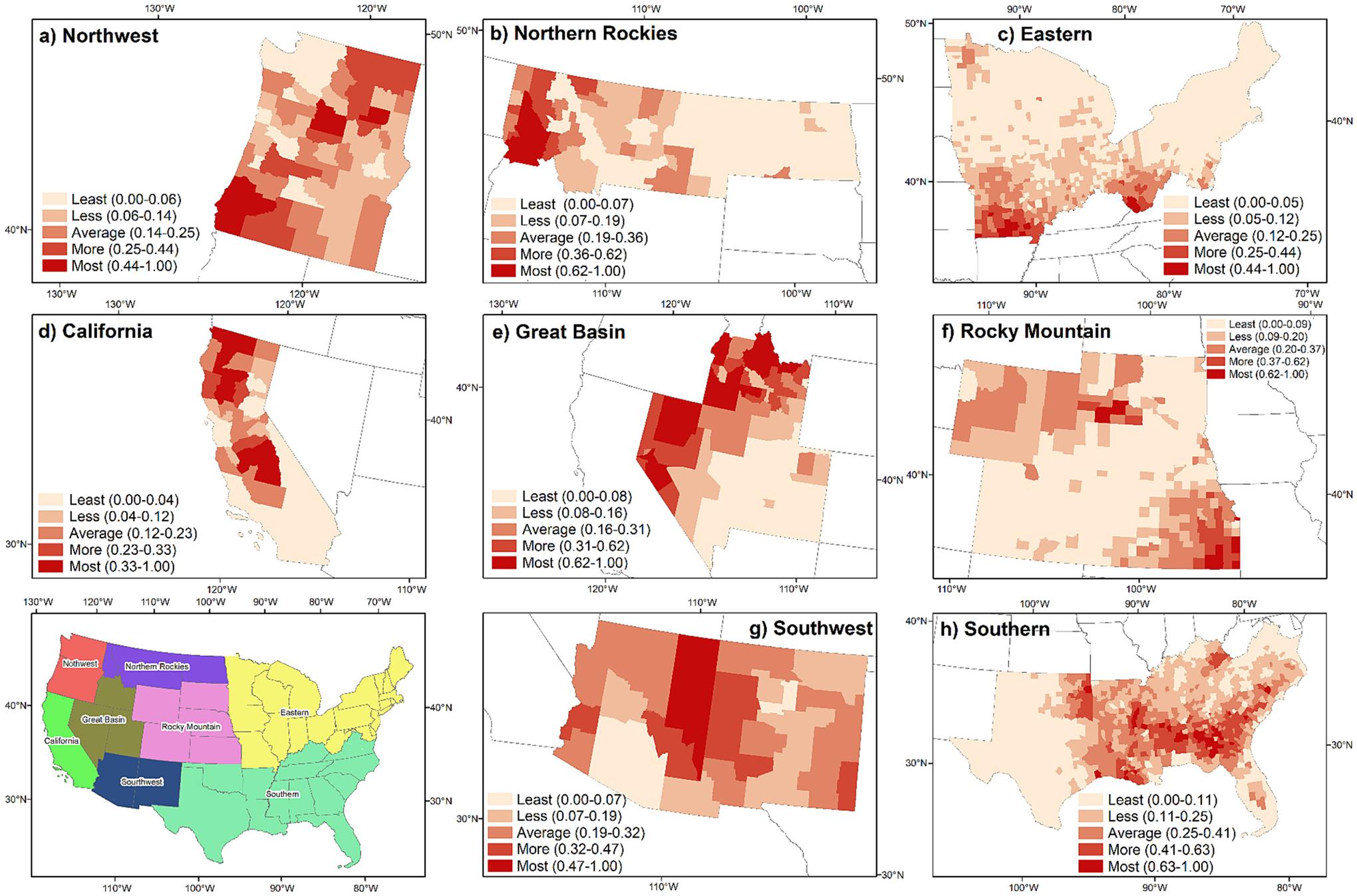
Eight regions’ community health vulnerability index classified into five categories using the natural Jenks method.

There was substantial variation within the US for the vulnerability index, which we show for eight regions where we separately calculated CHVI for each region (Fig. 4). Note that vulnerability index is a relative value that is not directly comparable between regions in Fig. 4. In other words, identical values in two distinct regions do not necessarily indicate equivalent levels of vulnerability as we have independently computed the vulnerability index for each region. We have thus used different breakpoints for each region which effectively represent the spatial patterns of vulnerability. Vulnerable areas that emerge include the southwestern and northeastern part of the Northwest region; western part of the Northern Rockies region; southern part of the Eastern region; northern and middle part of the California region; northwestern part of the Great basin region; northern and southeastern parts of the Rocky Mountain region; central part of the Southwest region; and central part of the Southern region.

### 3.2. Associations between exposure and the calculated indices and inequality in the indices

We investigated the relationships between fire-PM_2.5_ exposure and the calculated sub- and overall indices (i.e., adaptive capacity, sensitivity, and CHVI) using Spearman correlation coefficient. All of the indices were significantly associated with fire-PM_2.5_ exposure. The adaptive capacity sub-index (*r* = 0.45, 95 % CI: 0.41–0.48), the sensitivity sub-index (*r* = 0.47, 95 % CI: 0.45–0.50), and the CHVI (*r* = 0.87, 95 % CI: 0.86–0.88) were all positively associated with fire-PM_2.5_ exposure. This suggests that counties with lower adaptive capacity (higher adaptive capacity sub-index), higher sensitivity, and higher vulnerability tend to have higher fire-PM_2.5_ exposure. Figures on the relationships between exposure/CHVI and each sensitivity/adaptive capacity variable are in Supplementary Figs. S4 through S7.

We also tabulated the statistical summary of daily fire-PM_2.5_ exposures by each index’s decile (Supplementary Table S12). We overall observed that the counties with higher adaptive capacity (lower adaptive capacity sub-index), lower sensitivity, and lower vulnerability tended to have lower fire-PM_2.5_ exposure. In addition, we found that a higher percentage of counties were categorized into the highest daily fire-PM_2.5_ category (>1.50 μg/m^3^) when the adaptive capacity is low (when the adaptive capacity sub-index is high), sensitivity is high, and vulnerability is high. Here, unlike the sensitivity and adaptive capacity sub-indices, the vulnerability index was based on the exposure, which in turn, makes the association between them strong by the study design. A more detailed interpretation of this table can be found in Supplementary Material. In addition, we checked the inequality of the derived indices with Gini coefficients. Results show that the exposure sub-index (Gini coefficients: 0.35) and CHVI (0.54) were not evenly distributed compared to the adaptive capacity (0.23) and sensitivity sub-index (0.18).

### 3.3. Increases in unhealthy air quality days due to fire-PM_2.5_

We separately counted the annual number of days between 12 and 35 μg/m^3^ (moderate air quality days) and >35 μg/m^3^ (at or above unhealthy air quality days for sensitive groups) with both all-sources PM_2.5_ and all-sources PM_2.5_ except wildland fires, categorized by each index’s decile. The difference between the two model products is the contribution of fire-PM_2.5_ to the increases in the number of moderate and at or above unhealthy air quality days/year for sensitive groups.

**Fig. 5.**
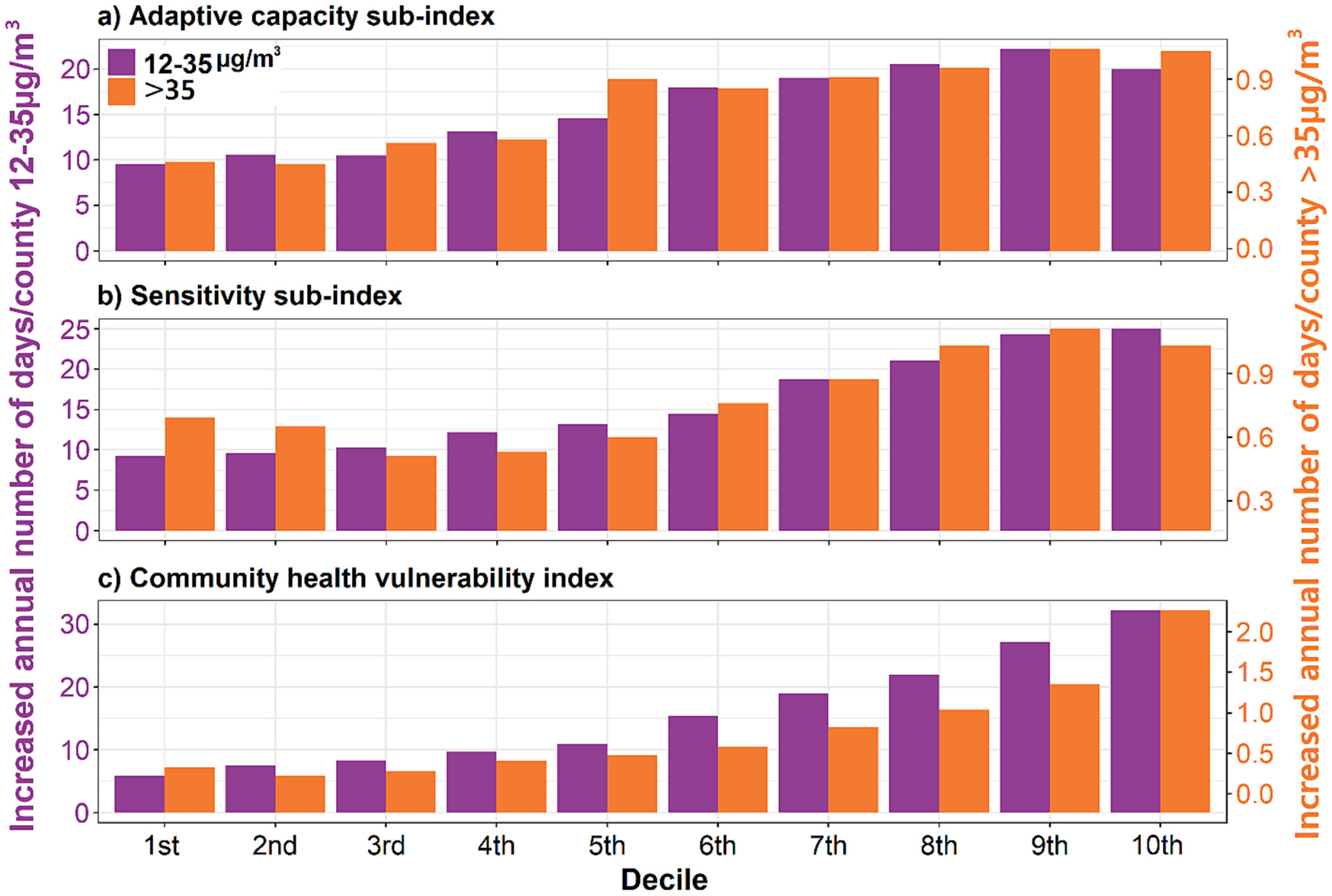
Increases in the number of days per year between 12 and 35 μg/m^3^ and >35 μg/m^3^ by decile of adaptive capacity sub-index, sensitivity sub-index, and community health vulnerability index due to wildland fires.

Generally, counties with lower adaptive capacity (higher adaptive capacity sub-index), higher sensitivity, and higher vulnerability tend to have a greater increase in the number of moderate and at or above unhealthy air quality days/year for sensitive groups (Fig. 5). For example, the increases in annual number of days between 12 and 35 μg/m^3^ and >35 μg/m^3^ respectively increased from 9.6 days (1st decile) to 20.0 days (10th decile) and from 0.5 days (1st decile) to 1.1 days (10th decile) with the increase in adaptive capacity sub-index. For the sensitivity sub-index, we also observed the increases in the annual number of days between 12 and 35 μg/m^3^ and >35 μg/m^3^ increased from 9.2 days (1st decile) to 25.1 days (10th decile) and from 0.7 days (1st decile) to 1.0 days (10th decile). For the CHVI, the increases in annual number of moderate air quality days and at or above unhealthy air quality days for sensitive groups respectively increased from 5.9 days (1st decile) to 32.2 days (10th decile) and from 0.3 days (1st decile) to 2.3 days (10th decile). More information on the number of days between 12 and 35 μg/m^3^ and >35 μg/m^3^ for all-sources PM_2.5_, all-sources PM_2.5_ without wildland fires, and the difference between the two data sets (impact of fire-PM_2.5_ on the increases in the number of moderate and at or above unhealthy air quality days/year for sensitive groups) can be found in Supplementary Tables S13–15.

### 3.4. Population size at risk

**Fig. 6.**
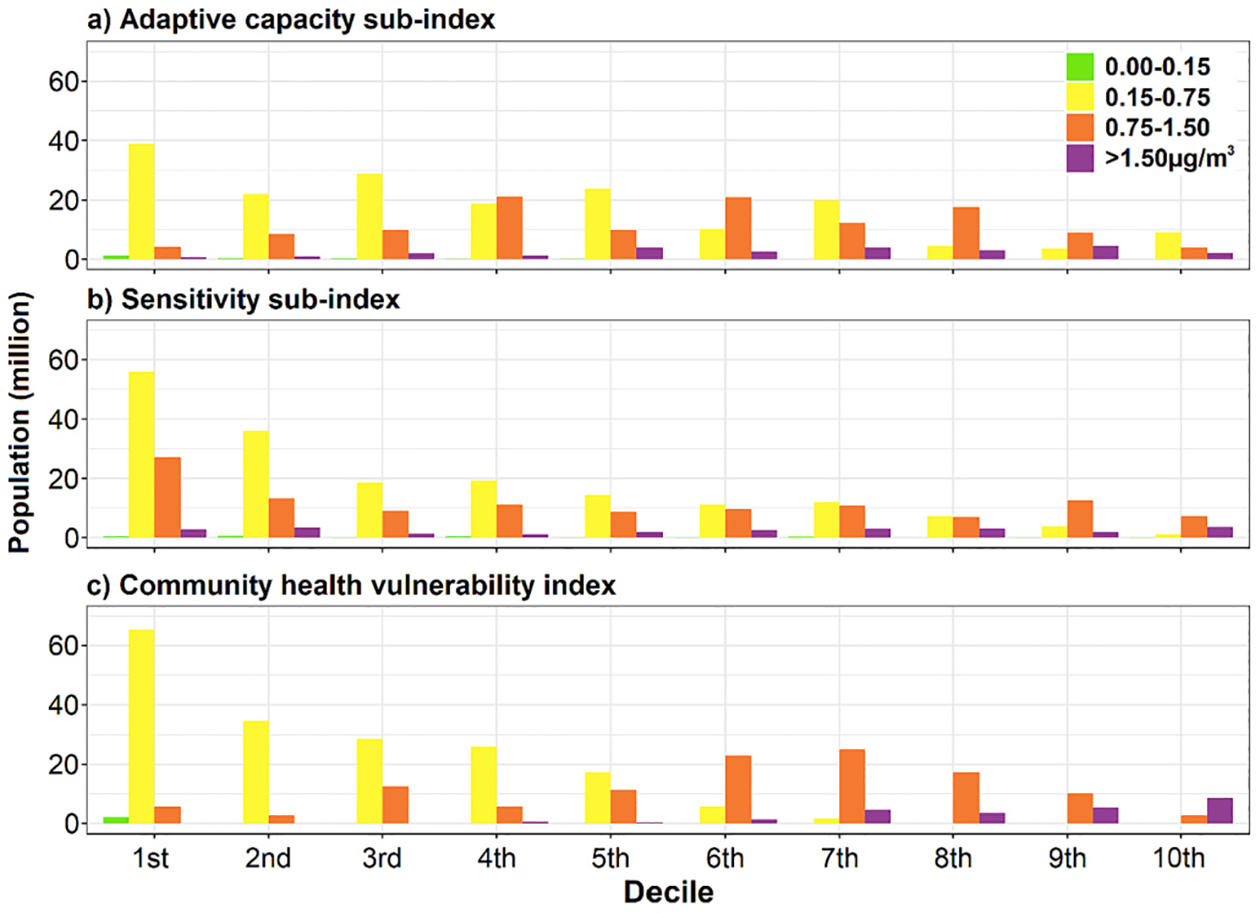
Population size by daily fire-PM_2.5_ concentrations threshold and decile for each index (adaptive capacity, sensitivity, and community health vulnerability index).

Fig. 6 shows the number of people at risk by daily fire-PM_2.5_ concentration and decile for multiple sub- and overall indices. Overall, counties with lower adaptive capacity (higher adaptive capacity sub-index), higher sensitivity, and higher vulnerability have small populations compared to their counterparts. More importantly, the counties with lower adaptive capacity (higher adaptive capacity sub-index), higher sensitivity, and higher vulnerability have a higher proportion of high daily fire-PM_2.5_ concentrations (i.e., 0.75–1.50, 1.50 μg/m^3^). Approximately, 44.9 million people have the highest adaptive capacity (1st decile) while 14.9 million people have the lowest adaptive capacity (10th decile). Among the 14.9 million people with the lowest adaptive capacity, 2.1 million people were also exposed to high daily fire-PM_2.5_ exposure (>1.50 μg/m^3^). For the sensitivity sub-index, 86.2 million people have the lowest sensitivity sub-index (1st decile) while 12.0 million people have the highest sensitivity (10th decile). One fourth of the 12.0 million people also experienced high daily fire-PM_2.5_ exposure (>1.50 μg/m^3^). For the CHVI, 73.3 million people have the lowest vulnerability index (1st decile) and 11.5 million people have the highest vulnerability (10th decile). Seventy-five percent of 11.5 million people were also exposed to high daily fire-PM_2.5_ concentration (>1.50 μg/m^3^). More details can be found in Supplementary Table S16. As a separate analysis, we further detected the counties experiencing high daily fire-PM_2.5_ concentration (>1.50 μg/m^3^), lowest adaptive capacity (>99th percentile), and highest sensitivity (>99th percentile). The results show that a total of five counties located in Alabama (Greene, Dallas Counties) and West Virginia (Boone, Logan, Mingo Counties) States correspond to these conditions, representing the highest risk (Supplementary Fig. S8).

## 4. Discussion

This study developed a measure of county-level community vulnerability for fire-PM_2.5_ based on adaptive capacity, sensitivity, and exposure at the national and regional level. This is one of several important extensions compared to other fire risk studies that do not focus on smoke/health and use CHVI broadly. We showed that the counties with higher daily fire-PM_2.5_ exposure tend to have lower adaptive capacity, higher sensitivity, and higher vulnerability at the county level. Our results complement previous studies that separately examined the impact of PM_2.5_ exposure (Kondo et al., 2022; Liu et al., 2015; Reid et al., 2016) and wildland fire smoke vulnerability (Rappold et al., 2017). These studies presented evidence that communities with higher PM_2.5_ exposure and higher vulnerability could be at higher risk of health risks resulting in increased smoke-related mortality and morbidity. Our results further described the relationships between fire-PM_2.5_ exposure levels and adaptive capacity, sensitivity, and CHVI. We found that counties with high fire-PM_2.5_ exposure may also be at higher risk as a result of corresponding low adaptive capacity and high sensitivity. This study bolsters recommendations that have argued for a multifaceted approach that addresses not only exposure, but also adaptive capacity and sensitivity, to reducing impacts from wildland fire smoke.

Relatedly, we discovered that counties with higher vulnerability are more likely to face a heightened risk of experiencing poor air quality during wildland fires compared to their counterparts. Previous studies have highlighted environmental inequality, asserting that marginalized communities, often characterized by low-income and minority populations, disproportionately bear the impact of natural disasters (Cutter, 1995; Institute of Medicine, 1999). Environmental inequality has been already identified in many vulnerability studies focusing on various environmental disasters such as heat waves (e.g., Estoque et al., 2020), floods (e.g., Maantay and Maroko, 2009), wildfires (e.g., Davies et al., 2018), and hurricanes (e.g., Bodenreider et al., 2019). These studies generally support the concept that socially and economically vulnerable communities are more likely to be exposed to higher risks of environmental hazards, such as air and water pollution and extreme weather events. Environmental inequality and environmental justice have recently garnered significant attention within the environmental discourse as efforts to mitigate the unequal distribution of exposure to environmental hazards (Whitehead, 2015). Multiple governments and agencies, including the EPA, CDC, and National Institute of Environmental Health Sciences (NIEHS), persist in their efforts to address disparities in environmental health through tools, collaborations, and public health initiatives. A vulnerability assessment could serve as an effective tool for identifying environmental inequality issues.

A large number of people are likely vulnerable to impacts of wildland fire smoke at least along one dimension of concern: 14.9, 12.0, and 11.5 million people have the lowest adaptive capacity (10th decile), the highest sensitivity (10th decile), and the highest vulnerability (10th decile), respectively. We showed that the spatial distribution of these populations was clustered: for example, five counties (total population: 127,285) located in Alabama (Greene, Dallas Counties) and West Virginia (Boone, Logan, Mingo Counties) experienced high average daily fire-PM_2.5_ concentrations (>1.5 μg/m^3^) and had the lowest adaptive capacity (>99th percentile), and the highest sensitivity (>99th percentile). Furthermore, the counties with lower adaptive capacity, higher sensitivity, and higher vulnerability often had a higher increase in the number of days between 12 and 35 μg/m^3^ (moderate air quality days) and >35 μg/m^3^ (at or above unhealthy air quality days for sensitive groups) due to fire-PM_2.5_ concentrations. For example, in the least vulnerable group (1st decile of the CHVI), fire-PM_2.5_-induced increases in the number of days between 12 and 35 μg/m^3^ and >35 μg/m^3^ were 5.9 and 0.3 days/year, respectively. On the other hand, the most vulnerable group (10th decile of the CHVI) experienced substantially higher increases of 32.2 and 2.3 days/year, which corresponds to approximately 5 to 6 times more days compared to the 1st decile of the CHVI.

Our results show additional vulnerable areas that were not captured with CHVI methods of Rappold et al. (2017). While Rappold et al. (2017) suggest that the Southern US had the highest vulnerability, our study showed two hot spots of high vulnerability: Northwestern (the Northwest and California regions) and Southern US. We suspect that this difference comes from at least two factors. First, while Rappold et al.’s (2017) vulnerability index is based on five years of data from 2008 to 2012, our study is based on eleven years of data from 2008 to 2018. Recent wildfires that occurred in the Western US could have potentially changed this pattern. Second, we surmise that a significant difference is that Rappold et al. (2017) did not incorporate adaptive capacity into their analysis. We believe presented work advances Rappold et al.’s (2017) CHVI by including the adaptive capacity. The rapidly changing nature of wildfires in the last decade alone in the western US (Dennison et al., 2014; Kramer et al., 2018) suggests employing longer time series could elucidate significant and important differences in vulnerable areas. Given this trend in wildfires is likely to continue in the future (Neumann et al., 2021), there will be a need to continually revisit and update vulnerability assessments to inform public health, fire management, and other decisions and priorities.

Our results also align with some of Rappold et al.’s (2017) results. We found the western US had longer and higher smoke PM_2.5_ exposure while the Eastern US experienced shorter and lower smoke PM_2.5_ exposure. Both Rappold et al. (2017) and our study observed high concentrations over mesic and dry mixed-conifer forested regions of Northern California and Pacific Northwest; within hardwood, pine and southern mixed forests; as well as in the wetlands across the Southeast. These spatial patterns are associated with the type of fires in these contexts: the majority of the emissions in the Southeast were smaller and more localized wildland fires (e.g., agricultural burning and prescribed burning), while wildland fires in the Western US are larger and longer lasting and can therefore increase the number of moderate and at or above unhealthy air quality days/year for sensitive groups (Rappold et al., 2017). This result suggests the potential benefits of applying tools such as prescribed burning and ecological thinning as options for managing fuels in the presence of inevitable wildfires (D’Evelyn et al., 2022).

Finally, our results support the broader literature showing the Southern US tends to have lower adaptive capacity to wildland fire smoke than the Northern US due to the distribution of race/ethnicity and socioeconomic status variables. Casey et al. (2017) showed similar spatial patterns representing higher proportions of non-White individuals, of individuals without a high school diploma, and of households below the federal poverty level in the Southern US. Jbaily et al. (2022) also reported that a higher proportion of non-White and low-income ZIP Code Tabulation Areas are concentrated in the Southern US. Our findings support the idea that communities with limited adaptive capacity should be supported in implementing protective measures to enhance resilience and reduce the negative health impacts of wildland fire smoke. These measures include both improved community fire mitigation practices and smoke risk communication (D’Evelyn et al., 2022; Rappold et al., 2017; Treves et al., 2022; Van Deventer et al., 2021; Wood et al., 2022).

We note several limitations that can be addressed by future analyses. Our analysis presents a critical first step in the analysis of vulnerability as it relates to broader societal well-being. However, the derived indices were not validated in this study, as the validation process is beyond the current scope of our research. Future work could examine the degree to which CHVI, as well as its sub-indices, are associated with relevant health outcomes. Previous vulnerability studies have used total property damage or the number of deaths/illness as a proxy for vulnerability to validate their indices (e.g., Ortega et al., 2019; Tellman et al., 2020). For our study, an ideal analysis would examine associations between the sub- and overall vulnerability indices and mortality or morbidity outcomes.

Second, CMAQ data, similar to most models, contains inherent biases (see, Appel et al., 2021; Wilkins et al., 2018). As those biases impact CHVI, our results indicate similar findings as Rappold et al. (2017) reported: 1) high biases at low PM_2.5_ concentrations which may come from too dispersive plumes and too high emission from small fires, 2) over-estimated small fire PM_2.5_ concentrations across all seasons, 3) limited model ability which does not simulate the smoldering aspects of peat fires well, and 4) incomplete emission data which excluded misspecified emissions in the emission inventory. Wilkins et al. (2018) also pointed out that the CMAQ model represents a low bias at higher emissions and a high bias at lower emissions. The model performs better for larger sources, meaning that our results for areas experiencing vulnerability from smaller fires could be biased lower than in reality. Further, how the model captures plume rise and dispersion could also alter the accuracy of this analysis due to incorrect placement of emissions.

Third, several uncertainties exist in the process of: 1) variable selection; 2) variable weighting; 3) variable standardization; 4) vulnerability calculation methodology; and 5) selecting the spatial resolution of variables. These and other uncertainties common in vulnerability assessments are further described in Tate (2013, 2012). Finally, variables estimated for different years and obtained from different sources could contribute uncertainty in the analysis. In particular, some variables based on the estimates such as adult asthma, pediatric asthma, and COPD could have high uncertainty and error.

## 5. Conclusions

The results presented here point to at least two practical implications. As fire-PM_2.5_ can travel over thousands of miles and adversely impact people living far from the fire origins (Matz et al., 2020; Sapkota et al., 2005), regional maps of spatial patterns of fire-PM_2.5_ exposure, adaptive capacity, sensitivity, and vulnerability indices can provide critical, localized information to state and county officials. This information may be utilized in making decisions regarding land management, public health, occupational health, and preparedness interventions and provide other services to vulnerable populations. Second, CHVI can raise awareness about risks to public health by providing information about the annual average number of moderate and at or above unhealthy air quality days for sensitive groups at the county level. Knowledge about the health risk of wildfire or prescribed fire smoke through effective outreach and communication could be especially useful for counties where fire smoke is less common. Local or state governments could use the information for promoting evidence-based and culturally tailored health interventions or individual protective behaviors (e.g., using a mask, low-cost home air filters) to avoid the adverse impact of fire smoke. The results can help broader efforts to more efficiently allocate resources and reduce exposure inequities.

Given that wildland fires and smoke are likely to continue to increase in the future, we would expect future increases in morbidity and mortality caused by wildland fire smoke exposure without additional prevention/mitigation (Neumann et al., 2021). Identifying counties that are more vulnerable to wildland fire smoke, and the drivers of these vulnerabilities, is an important step in helping researchers and practitioners better understand, prepare for, and respond to future wildfire events.

## Supplementary Material

Supplement1

## Data Availability

The data used in the paper is all public data. All links for data downloads are in the manuscript.

## HIGHLIGHTS

- This study updates the community health vulnerability index for wildland fire smoke.
- Counties with higher PM_2.5_ tend to show lower adaptive capacity and higher sensitivity.
- Counties with higher vulnerability tend to experience higher PM_2.5_.
- Highly vulnerable counties have a higher increase in yearly moderate/unhealthy air days.
- Northwest, Northern Rockies, and Southern regions have higher vulnerability.
