# Supplementary material for: Advancing the community health vulnerability index for wildland fire smoke exposure
Source: Sci Total Environ. Author manuscript; Available in PMC 2025 May 19. (PMC12087597; doi:10.1016/j.scitotenv.2023.167834)
Supplement: Supplement1 [file NIHMS2073167-supplement-Supplement1.docx]

**Advancing the Community Health Vulnerability Index for Wildland Fire Smoke Exposure**

Jihoon Jung^a*^, Joseph L Wilkins^b,c^, Claire L Schollaert^d^, Yuta J Masuda^e^, John C. Flunker^d^, Rachel E Connolly^f^, Savannah M D’Evelyn^d^, Eimy Bonillia^b^, Ana G Rappold^g^, Ryan D Haugo^h^, Miriam E Marlier^f^, June T Spector^d^

^a^ Department of City and Regional Planning, University of North Carolina, Chapel Hill, NC, USA

^b^ Interdisciplinary Studies Department, Howard University, Washington, DC, USA

^c^ School of Environmental and Forest Sciences, University of Washington, Seattle, WA, USA

^d^ Department of Environmental & Occupational Health Sciences, University of Washington, Seattle, WA, USA

^e^ Partnerships and Programs, Vulcan LLC, Seattle, WA, USA

^f^ Department of Environmental Health Sciences, Jonathan and Karin Fielding School of Public Health, University of California, Los Angeles, CA USA

^g^ United States Environmental Protection Agency, Durham, NC, USA

^h^ The Nature Conservancy, Portland, OR, United States of America

* Corresponding author: Jihoon Jung

Email: [climategeo@gmail.com](mailto:climategeo@gmail.com)

Full postal address: New East Building, CB3140, Chapel Hill, NC 27599, US

**Supplementary material**

**Details on the general Community Multiscale Air Quality configuration**

Based on availability for a given year, various sources for fire emissions are aggregated into the inventory for each year in SMARTFIRE versions 2 – common input sources, Geospatial Multi-Agency Coordination (GeoMac) Perimeters, Incident Status Summary (ICS-209) reports, Hazard Mapping System (HMS) fire detects, and Monitoring Trends in Burn Severity (MTBS). Wildland fire emissions inventories estimated each year since 2002 are made available at ftp://ftp.epa.gov/EmisInventory/fires. The fire emission estimates used in this analysis were based on the following components from the BlueSky framework: SMARTFIRE2 (Sullivan et al., 2008) was used to reconcile the sources of fire activity data. Raffuse et al. (2012, 2007) describe the SMARTFIRE2 process of reconciling fires from ICS-209 reports. Once the fire is reconciled, the biomass fuel consumption was calculated using the US Forest Service’s CONSUME ver. 3.0 fuel consumption model (https://www.fs.fed.us/pnw/fera/fft/consumemodule.shtml) and the Fuel Characteristic Classification System (FCCS) fuel-loading database in the BlueSky Framework (Ottmar et al., 2007). Emission factors were taken from the Fire Emission Production Simulator (FEPS) model (<https://www.fs.fed.us/pnw/fera/feps>).

**References**

Ottmar, R.D., Sandberg, D. V, Riccardi, C.L., Prichard, S.J., 2007. An overview of the fuel characteristic classification system—quantifying, classifying, and creating fuelbeds for resource planning. Canadian Journal of Forest Research 37, 2383–2393.

Raffuse, S.M., Larkin, N.K., Lahm, P.W., Du, Y., 2012. Development of version 2 of the wildland fire portion of the National Emissions Inventory, in: 20th International Emission Inventory Conference, Tampa, Florida.

Raffuse, S.M., Sullivan, D.C., Chinkin, L.R., Pryden, D.A., Wheeler, N.J., Larkin, N.K., Soja, A., 2007. Integration and reconciliation of satellite-detected and Incident Command-reported wildfire information in the BlueSky Smoke Modeling Framework, in: Proceedings of the 6th Annual CMAS Conference. pp. 1–3.

Sullivan, D.C., Raffuse, S.M., Pryden, D.A., Craig, K.J., Reid, S.B., Wheeler, N.J.M., Chinkin, L.R., Larkin, N.K., Solomon, R., Strand, T., 2008. Development and applications of systems for modeling emissions and smoke from fires: the BlueSky smoke modeling framework and SMARTFIRE, in: 17th International Emissions Inventory Conference. pp. 2–5.

**A more detailed interpretation on Supplementary Table 8**

We also tabulated the statistical summary of daily fire-PM_2.5_ exposures by each index’s decile (Supplementary Table 8). For the adaptive capacity sub-index, we observed that counties with higher adaptive capacity tended to have lower fire-PM_2.5_ exposure. For example, the daily average fire-PM_2.5_ exposure of counties within the 1st decile (highest adaptive capacity) was 0.54μg/m^3^ whereas daily average fire-PM_2.5_ exposure of counties within the 10th decile (lowest adaptive capacity) for adaptive capacity sub-index was 1.14μg/m^3^. In addition, we observed a higher percentage of counties being categorized into the highest daily fire-PM_2.5_ category (>1.50μg/m^3^) when the adaptive capacity sub-index is low. For instance, while 28.9% of counties within the 10th decile (lowest adaptive capacity) were in the highest daily fire-PM_2.5_ category (>1.50μg/m^3^), only 1.3% of counties within the 1st decile (highest adaptive capacity) were in the highest daily fire-PM_2.5_ category (>1.50μg/m^3^).

For the sensitivity sub-index, we observed that daily fire-PM_2.5_ exposure increased with incremental increases in sensitivity sub-index (Supplementary Table 8). While the average exposure of counties within the 1st decile was 0.65μg/m^3^, the daily exposure of the most sensitive counties within the 10th decile was 1.27μg/m^3^. Similarly, the percentage of counties in the highest daily fire-PM_2.5_ category (>1.50μg/m^3^) increased with the increase in sensitivity sub-index decile (sensitivity sub-index deciles: 1st: 5.5%; 2nd:6.4%, 3rd: 4.5%; 4th: 4.5%; 5th: 6.8%; 6th: 10.0%; 7th: 17.7%; 8th: 24.1%; 9th: 23.8%; 10th: 29.3%).

For the CHVI index, counties with higher vulnerability index had higher daily fire-PM2.5 concentration (Supplementary Table 8). Unlike the sensitivity and adaptive capacity sub-indices, the vulnerability index was based on the exposure, which in turn, makes the association between them strong by the study design. The average fire-PM_2.5_ concentration of counties within the 10th decile vulnerability index (1.82μg/m^3^) was six times higher than the average concentration of counties within the 1st decile vulnerability index (0.37μg/m^3^). In addition, while most of the counties (71.1%) within the 10th decile vulnerability index was in the highest daily fire-PM_2.5_ category (>1.50μg/m^3^), no counties within the 1st decile vulnerability index were in the highest fire-PM_2.5_ category (>1.50μg/m^3^).

Supplementary Table S1. Data sources used for the CMAQ wildland fire model runs.

| Year | NEI year | CMAQ version | BEIS version | EGU CEM data | Gas phase chemistry | PM chemistry | Boundary inflow | WRF version |
| --- | --- | --- | --- | --- | --- | --- | --- | --- |
| 2008 | 2008 NEI | v5.0.1 | 3.14 | 2008 | CB05 | AERO6 | GEOS-CHEM | v3.4 |
| 2009 | 2008 NEI | v5.0.1 | 3.14 | 2009 | CB05 | AERO6 | GEOS-CHEM | v3.4 |
| 2010 | 2008 NEI | v5.0.1 | 3.14 | 2010 | CB05 | AERO6 | GEOS-CHEM | v3.4 |
| 2011 | 2011 NEI | v5.0.1 | 3.14 | 2011 | CB05 | AERO6 | GEOS-CHEM | v3.4 |
| 2012 | 2011 NEI | v5.0.2 | 3.14 | 2012 | CB05 | AERO6 | GEOS-CHEM | v3.4 |
| 2013 | 2011NEIv2 | v5.2 | 3.6.1 | 2013 | CB6r3 | AERO6 | GEOS-CHEM | v3.8 |
| 2014 | 2014NEIv1 | v5.2 | 3.6.1 | 2014 | CB6r3 | AERO6 | GEOS-CHEM | v3.8.1 |
| 2015 | 2014NEIv2 | v5.2.1 | 3.6.1 | 2015 | CB6r3 | AERO6 | Hemispheric CMAQ | v3.8.1 |
| 2016 | 2014NEIv2 | v5.2.1 | 3.6.1 | 2016 | CB6r3 | AERO7 | Hemispheric CMAQ | v3.8.1 |
| 2017 | 2014NEIv2 | v5.2.1 | 3.6.1 | 2017 | CB6r3 | AERO7 | Hemispheric CMAQ | v3.8.1 |
| 2018 | 2014NEIv2 | v5.3 | 3.6.1 | 2018 | CB6r3 | AERO7 | Hemispheric CMAQ | v3.8.1 |

Supplementary Table S2. Variables used for sensitivity (ACS: American Community Survey, CDC: Centers for Disease Control and Prevention).

| Variable | year | Unit | Direction | Data source |
| --- | --- | --- | --- | --- |
| Young population (<5) | 2019 | % | + | ACS |
| Elderly population (≥65) | 2019 |  |  |  |
| Agricultural and construction workers | 2019 |  |  |  |
| Diabetes | 2019 |  |  | CDC |
| Obesity | 2019 |  |  |  |
| Hypertension | 2017 |  |  |  |
| Adult asthma | 2020 |  |  |  |
| COPD | 2020 |  |  |  |
| Pediatric asthma | 2021 |  |  | American lung association |

Supplementary Table S3. Descriptive summary of sensitivity variables. Note that the average of these variables at the county level is not equivalent to the average for the entire country.

| Variables | Unit | Mean | SD | Max | Min |
| --- | --- | --- | --- | --- | --- |
| Young population (<5) | % | 5.8 | 1.3 | 21.4 | 0.4 |
| Elderly population (≥65) |  | 18.8 | 4.7 | 56.7 | 3.2 |
| Agricultural and construction workers |  | 8.5 | 3.6 | 46.0 | 0.0 |
| Diabetes |  | 8.7 | 1.6 | 17.6 | 4.1 |
| Obesity |  | 28.5 | 4.8 | 45.4 | 13.9 |
| Hypertension |  | 37.2 | 5.5 | 56.9 | 18.7 |
| Adult asthma |  | 10.1 | 0.9 | 14.1 | 7.1 |
| Pediatric asthma |  | 7.2 | 1.6 | 11.5 | 4.4 |
| COPD |  | 7.2 | 1.7 | 16.5 | 2.6 |

Supplementary Table S4. Variables used for adaptive capacity (ACS: American Community Survey, CDC: Centers for Disease Control and Prevention).

| Variable | Year | Unit | Direction | Data source |
| --- | --- | --- | --- | --- |
| Population in workforce | 2019 | % | − | ACS |
| Disabled population | 2019 |  | + |  |
| Black | 2019 |  |  |  |
| Hispanic | 2019 |  |  |  |
| American Indian and Alaska Native | 2019 |  |  |  |
| Single parent households | 2019 |  |  |  |
| Population without high school diploma | 2019 |  |  |  |
| Population speaking English less than well | 2019 |  |  |  |
| Below poverty households | 2019 |  |  |  |
| Unemployment rate | 2019 |  |  |  |
| Mobile home | 2019 |  |  |  |
| Multi-housing unit | 2019 |  |  |  |
| Households without car | 2019 |  |  |  |
| Population without health insurance | 2018 |  |  | CDC |
| Household income | 2019 | $ | − | ACS |
| Road density | 2018 | km/km^2^ |  | Meijer et al. (2018) |
| Hospitals | 2017 | Count |  | CDC |
| Pharmacies and drug stores | 2018 |  |  |  |
| Healthcare support occupations | 2019 | % |  | ACS |
| Protective service occupations  (Firefighting and prevention, And Other Protective Service Workers Including Supervisors) | 2019 |  |  |  |

Meijer, J. R., Huijbregts, M. A. J., Schotten, K. C. G. J., & Schipper, A. M. (2018). Global patterns of current and future road infrastructure. *Environmental Research Letters*, *13*(6), 064006.

Supplementary Table S5. Descriptive summary of adaptive capacity variables. Note that the average of these variables at the county level is not equivalent to the average for the entire country.

| Variables | Unit | Mean | SD | Max | Min |
| --- | --- | --- | --- | --- | --- |
| Population in workforce | % | 80.3 | 3.2 | 98.5 | 62.5 |
| Disabled population |  | 16.0 | 4.4 | 36.8 | 4.0 |
| Black |  | 9.1 | 14.5 | 87.2 | 0.0 |
| Hispanic |  | 9.4 | 13.9 | 99.2 | 0.0 |
| American Indian and Alaska Native |  | 2.0 | 7.8 | 93.3 | 0.0 |
| Single parent households |  | 15.7 | 5.1 | 55.3 | 0.0 |
| Population without high school diploma |  | 13.1 | 6.3 | 73.6 | 1.1 |
| Population speaking English less than well |  | 1.7 | 2.8 | 34.4 | 0.0 |
| Below poverty households |  | 15.1 | 6.3 | 55.5 | 2.4 |
| Unemployment rate |  | 5.2 | 2.7 | 30.2 | 0.0 |
| Mobile home |  | 12.0 | 9.3 | 55.9 | 0.0 |
| Multi-housing unit |  | 5.2 | 5.5 | 93.4 | 0.0 |
| Households without car |  | 6.3 | 4.5 | 88.0 | 0.0 |
| Population without health insurance |  | 11.5 | 5.1 | 32.2 | 2.4 |
| Healthcare support occupations |  | 3.6 | 1.6 | 16.9 | 0.0 |
| Protective service occupations  (Firefighting and prevention, And Other Protective Service Workers Including Supervisors) |  | 1.0 | 0.7 | 9.8 | 0.0 |
| Household income | $ | 27,672.7 | 5,895.1 | 70,390.0 | 8,641.0 |
| Road density | km/km^2^ | 738.6 | 1630.8 | 22630.3 | 0.0 |
| Hospitals | Count | 1.4 | 2.5 | 78.0 | 0.0 |
| Pharmacies and drug stores |  | 11.0 | 11.2 | 111.8 | 0.0 |

Supplementary Table S6. Descriptive summary of exposure levels and the number of days between 12 and 35μg/m^3^ (above moderate air) and >35μg/m^3^ (at or above unhealthy for sensitive groups) by region.

|  | All-sources-PM_2.5_ (μg/m^3^) | | | Fire-PM_2.5_ (μg/m^3^) | | |
| --- | --- | --- | --- | --- | --- | --- |
|  | Average  level | Average number of days per year between 12-35 | Average Number of days per year >35 | Average  level | Average Number of days per year between 12-35 | Average Number of days per year >35 |
| Northwest | 4.36 | 16.98 | 2.39 | 1.35 | 6.15 | 1.88 |
| Northern Rockies | 3.69 | 7.55 | 1.04 | 0.80 | 2.63 | 0.90 |
| Eastern | 7.90 | 63.01 | 0.91 | 0.61 | 1.23 | 0.06 |
| California | 6.27 | 29.39 | 4.55 | 1.97 | 7.84 | 3.62 |
| Great Basin | 3.16 | 7.34 | 0.81 | 0.79 | 3.55 | 0.65 |
| Rocky Mountain | 4.73 | 16.17 | 0.21 | 0.61 | 1.37 | 0.11 |
| Southwest | 3.03 | 1.47 | 0.06 | 0.30 | 0.26 | 0.03 |
| Southern | 7.49 | 50.59 | 1.02 | 1.16 | 3.77 | 0.30 |

Supplementary Table S7. Descriptive summary by region. Note that the average of these variables at the county level is not equivalent to the average for the entire country.

|  | Adaptive capacity sub-index | Sensitivity sub-index | Community health vulnerability index |
| --- | --- | --- | --- |
| Northwest | 0.31 | 0.44 | 0.16 |
| Northern Rockies | 0.26 | 0.38 | 0.07 |
| Eastern | 0.25 | 0.42 | 0.06 |
| California | 0.33 | 0.28 | 0.18 |
| Great Basin | 0.34 | 0.38 | 0.09 |
| Rocky Mountain | 0.28 | 0.37 | 0.05 |
| Southwest | 0.49 | 0.43 | 0.04 |
| Southern | 0.43 | 0.52 | 0.24 |

Supplementary Table S8. The 100 most vulnerable counties using the multiplicative method. Counties identified as the 100 most vulnerable counties from both multiplicative and additive methods are in bold.

|  | State | County | Adaptive capacity | Sensitivity | exposure | vulnerability |
| --- | --- | --- | --- | --- | --- | --- |
| 1 | **Georgia** | **Clay** | 0.75 | 0.71 | 0.36 | 0.19 |
| 2 | **Alabama** | **Barbour** | 0.63 | 0.72 | 0.40 | 0.18 |
| 3 | **Louisiana** | **Assumption** | 0.53 | 0.63 | 0.52 | 0.17 |
| 4 | **Georgia** | **Dougherty** | 0.60 | 0.68 | 0.43 | 0.17 |
| 5 | **Georgia** | **Terrell** | 0.65 | 0.66 | 0.40 | 0.17 |
| 6 | **Georgia** | **Quitman** | 0.74 | 0.64 | 0.36 | 0.17 |
| 7 | **Alabama** | **Macon** | 0.62 | 0.66 | 0.40 | 0.17 |
| 8 | **Alabama** | **Lowndes** | 0.74 | 0.79 | 0.28 | 0.17 |
| 9 | **Alabama** | **Bullock** | 0.64 | 0.64 | 0.40 | 0.17 |
| 10 | **Oklahoma** | **Adair** | 0.60 | 0.78 | 0.35 | 0.17 |
| 11 | **Georgia** | **Randolph** | 0.62 | 0.71 | 0.38 | 0.16 |
| 12 | **Georgia** | **Baker** | 0.61 | 0.61 | 0.44 | 0.16 |
| 13 | **Alabama** | **Dallas** | 0.62 | 0.90 | 0.28 | 0.16 |
| 14 | **Georgia** | **Calhoun** | 0.70 | 0.55 | 0.40 | 0.16 |
| 15 | **Alabama** | **Wilcox** | 0.69 | 0.79 | 0.28 | 0.15 |
| 16 | **Mississippi** | **Holmes** | 0.80 | 0.86 | 0.22 | 0.15 |
| 17 | **Georgia** | **Mitchell** | 0.61 | 0.59 | 0.41 | 0.15 |
| 18 | **Georgia** | **Colquitt** | 0.67 | 0.60 | 0.37 | 0.15 |
| 19 | **Florida** | **Gadsden** | 0.58 | 0.71 | 0.36 | 0.15 |
| 20 | **Georgia** | **Macon** | 0.61 | 0.68 | 0.35 | 0.14 |
| 21 | **Georgia** | **Grady** | 0.57 | 0.58 | 0.44 | 0.14 |
| 22 | **Alabama** | **Greene** | 0.67 | 0.83 | 0.26 | 0.14 |
| 23 | **Georgia** | **Early** | 0.60 | 0.67 | 0.36 | 0.14 |
| 24 | **Georgia** | **Atkinson** | 0.78 | 0.59 | 0.31 | 0.14 |
| 25 | **Georgia** | **Crisp** | 0.64 | 0.66 | 0.33 | 0.14 |
| 26 | **Oregon** | **Josephine** | 0.33 | 0.53 | 0.81 | 0.14 |
| 27 | **West Virginia** | **Mingo** | 0.50 | 0.99 | 0.28 | 0.14 |
| 28 | **Alabama** | **Russell** | 0.51 | 0.66 | 0.41 | 0.14 |
| 29 | **Mississippi** | **Noxubee** | 0.69 | 0.76 | 0.26 | 0.13 |
| 30 | **Texas** | **Jefferson** | 0.45 | 0.57 | 0.52 | 0.13 |
| 31 | **California** | **Del Norte** | 0.44 | 0.30 | 1.00 | 0.13 |
| 32 | **Georgia** | **Sumter** | 0.55 | 0.64 | 0.37 | 0.13 |
| 33 | **Georgia** | **Taylor** | 0.61 | 0.60 | 0.36 | 0.13 |
| 34 | **Georgia** | **Dooly** | 0.62 | 0.62 | 0.34 | 0.13 |
| 35 | **South Carolina** | **Allendale** | 0.71 | 0.63 | 0.29 | 0.13 |
| 36 | **Georgia** | **Decatur** | 0.48 | 0.66 | 0.41 | 0.13 |
| 37 | **Georgia** | **Warren** | 0.67 | 0.63 | 0.30 | 0.13 |
| 38 | **Oklahoma** | **Cherokee** | 0.52 | 0.71 | 0.35 | 0.13 |
| 39 | Georgia | Thomas | 0.50 | 0.54 | 0.47 | 0.13 |
| 40 | **Georgia** | **Jenkins** | 0.61 | 0.67 | 0.31 | 0.13 |
| 41 | **Louisiana** | **Iberia** | 0.49 | 0.66 | 0.39 | 0.13 |
| 42 | **Mississippi** | **Washington** | 0.68 | 0.91 | 0.20 | 0.12 |
| 43 | **Alabama** | **Hale** | 0.61 | 0.77 | 0.26 | 0.12 |
| 44 | **Georgia** | **Turner** | 0.61 | 0.61 | 0.33 | 0.12 |
| 45 | **Georgia** | **Stewart** | 0.81 | 0.41 | 0.36 | 0.12 |
| 46 | **Mississippi** | **Coahoma** | 0.65 | 0.91 | 0.20 | 0.12 |
| 47 | **Alabama** | **Sumter** | 0.64 | 0.70 | 0.27 | 0.12 |
| 48 | **Georgia** | **Taliaferro** | 0.64 | 0.63 | 0.30 | 0.12 |
| 49 | **Georgia** | **Johnson** | 0.62 | 0.60 | 0.33 | 0.12 |
| 50 | **Georgia** | **Clinch** | 0.67 | 0.65 | 0.28 | 0.12 |
|  | State | County | Adaptive capacity | Sensitivity | exposure | vulnerability |
| 51 | **Mississippi** | **Kemper** | 0.63 | 0.71 | 0.27 | 0.12 |
| 52 | **Mississippi** | **Leake** | 0.68 | 0.73 | 0.24 | 0.12 |
| 53 | **Georgia** | **Jefferson** | 0.58 | 0.65 | 0.32 | 0.12 |
| 54 | **Georgia** | **Treutlen** | 0.63 | 0.59 | 0.32 | 0.12 |
| 55 | **Oklahoma** | **Sequoyah** | 0.50 | 0.71 | 0.33 | 0.12 |
| 56 | **Mississippi** | **Bolivar** | 0.64 | 0.86 | 0.22 | 0.12 |
| 57 | **North Carolina** | **Robeson** | 0.66 | 0.93 | 0.19 | 0.12 |
| 58 | **Mississippi** | **Scott** | 0.66 | 0.75 | 0.23 | 0.12 |
| 59 | Georgia | Talbot | 0.53 | 0.61 | 0.36 | 0.12 |
| 60 | **Alabama** | **Marengo** | 0.52 | 0.79 | 0.28 | 0.11 |
| 61 | Georgia | Muscogee | 0.47 | 0.62 | 0.39 | 0.11 |
| 62 | **Georgia** | **Laurens** | 0.50 | 0.72 | 0.32 | 0.11 |
| 63 | Georgia | Brooks | 0.51 | 0.58 | 0.39 | 0.11 |
| 64 | **Mississippi** | **Jefferson** | 0.72 | 0.76 | 0.21 | 0.11 |
| 65 | **Alabama** | **Conecuh** | 0.57 | 0.70 | 0.29 | 0.11 |
| 66 | Louisiana | St. Mary | 0.45 | 0.70 | 0.36 | 0.11 |
| 67 | Georgia | Candler | 0.62 | 0.59 | 0.31 | 0.11 |
| 68 | Georgia | Telfair | 0.55 | 0.66 | 0.31 | 0.11 |
| 69 | **Mississippi** | **Humphreys** | 0.79 | 0.76 | 0.19 | 0.11 |
| 70 | **Alabama** | **Perry** | 0.51 | 0.80 | 0.28 | 0.11 |
| 71 | Alabama | Tallapoosa | 0.46 | 0.68 | 0.36 | 0.11 |
| 72 | Oklahoma | Latimer | 0.51 | 0.68 | 0.33 | 0.11 |
| 73 | Georgia | Ben Hill | 0.56 | 0.62 | 0.32 | 0.11 |
| 74 | Georgia | Cook | 0.55 | 0.60 | 0.34 | 0.11 |
| 75 | Georgia | Webster | 0.57 | 0.54 | 0.36 | 0.11 |
| 76 | Georgia | Marion | 0.53 | 0.56 | 0.37 | 0.11 |
| 77 | Georgia | Wheeler | 0.60 | 0.58 | 0.32 | 0.11 |
| 78 | **Alabama** | **Monroe** | 0.54 | 0.75 | 0.27 | 0.11 |
| 79 | Georgia | Emanuel | 0.55 | 0.64 | 0.31 | 0.11 |
| 80 | **West Virginia** | **Logan** | 0.45 | 0.99 | 0.25 | 0.11 |
| 81 | **Oklahoma** | **McIntosh** | 0.51 | 0.79 | 0.27 | 0.11 |
| 82 | **Oklahoma** | **Delaware** | 0.50 | 0.75 | 0.29 | 0.11 |
| 83 | **California** | **Trinity** | 0.33 | 0.38 | 0.86 | 0.11 |
| 84 | **Mississippi** | **Neshoba** | 0.57 | 0.77 | 0.25 | 0.11 |
| 85 | **Kentucky** | **Breathitt** | 0.59 | 0.73 | 0.25 | 0.11 |
| 86 | Georgia | Wilcox | 0.54 | 0.61 | 0.33 | 0.11 |
| 87 | Florida | Hamilton | 0.68 | 0.58 | 0.27 | 0.11 |
| 88 | **Mississippi** | **Panola** | 0.56 | 0.83 | 0.23 | 0.11 |
| 89 | Georgia | Meriwether | 0.54 | 0.61 | 0.33 | 0.11 |
| 90 | Oklahoma | Haskell | 0.50 | 0.68 | 0.31 | 0.11 |
| 91 | **Mississippi** | **Claiborne** | 0.78 | 0.66 | 0.21 | 0.11 |
| 92 | **South Carolina** | **Williamsburg** | 0.60 | 0.80 | 0.22 | 0.11 |
| 93 | **Alabama** | **Clarke** | 0.51 | 0.78 | 0.26 | 0.11 |
| 94 | Georgia | Twiggs | 0.54 | 0.59 | 0.33 | 0.11 |
| 95 | Florida | Madison | 0.55 | 0.62 | 0.31 | 0.11 |
| 96 | Alabama | Butler | 0.50 | 0.73 | 0.29 | 0.11 |
| 97 | Alabama | Choctaw | 0.54 | 0.73 | 0.27 | 0.11 |
| 98 | Alabama | Talladega | 0.49 | 0.72 | 0.29 | 0.11 |
| 99 | **Mississippi** | **Winston** | 0.55 | 0.74 | 0.26 | 0.11 |
| 100 | Georgia | Washington | 0.59 | 0.55 | 0.32 | 0.11 |

Supplementary Table S9. The 100 most vulnerable counties using the additive method. Counties identified as the 100 most vulnerable counties from both multiplicative and additive methods are in bold.

|  | State | County | Adaptive capacity | Sensitivity | exposure | vulnerability |
| --- | --- | --- | --- | --- | --- | --- |
| 1 | South Dakota | Oglala Lakota | 0.98 | 0.90 | 0.09 | 1.97 |
| 2 | **Mississippi** | **Holmes** | 0.80 | 0.86 | 0.22 | 1.88 |
| 3 | **Georgia** | **Clay** | 0.75 | 0.71 | 0.36 | 1.83 |
| 4 | **Alabama** | **Lowndes** | 0.74 | 0.79 | 0.28 | 1.81 |
| 5 | **Alabama** | **Dallas** | 0.62 | 0.90 | 0.28 | 1.81 |
| 6 | **Mississippi** | **Washington** | 0.68 | 0.91 | 0.20 | 1.78 |
| 7 | **North Carolina** | **Robeson** | 0.66 | 0.93 | 0.19 | 1.78 |
| 8 | South Dakota | Todd | 0.85 | 0.86 | 0.07 | 1.77 |
| 9 | **Mississippi** | **Coahoma** | 0.65 | 0.91 | 0.20 | 1.77 |
| 10 | **West Virginia** | **Mingo** | 0.50 | 0.99 | 0.28 | 1.77 |
| 11 | **Alabama** | **Wilcox** | 0.69 | 0.79 | 0.28 | 1.76 |
| 12 | **Alabama** | **Greene** | 0.67 | 0.83 | 0.26 | 1.76 |
| 13 | **Alabama** | **Barbour** | 0.63 | 0.72 | 0.40 | 1.75 |
| 14 | **California** | **Del Norte** | 0.44 | 0.30 | 1.00 | 1.75 |
| 15 | **Mississippi** | **Humphreys** | 0.79 | 0.76 | 0.19 | 1.74 |
| 16 | **Georgia** | **Quitman** | 0.74 | 0.64 | 0.36 | 1.74 |
| 17 | **Oklahoma** | **Adair** | 0.60 | 0.78 | 0.35 | 1.73 |
| 18 | West Virginia | McDowell | 0.54 | 1.00 | 0.19 | 1.73 |
| 19 | **Georgia** | **Terrell** | 0.65 | 0.66 | 0.40 | 1.71 |
| 20 | **Mississippi** | **Bolivar** | 0.64 | 0.86 | 0.22 | 1.71 |
| 21 | **Georgia** | **Dougherty** | 0.60 | 0.68 | 0.43 | 1.71 |
| 22 | **Mississippi** | **Noxubee** | 0.69 | 0.76 | 0.26 | 1.71 |
| 23 | **Georgia** | **Randolph** | 0.62 | 0.71 | 0.38 | 1.70 |
| 24 | **Mississippi** | **Jefferson** | 0.72 | 0.76 | 0.21 | 1.69 |
| 25 | **West Virginia** | **Logan** | 0.45 | 0.99 | 0.25 | 1.69 |
| 26 | **Alabama** | **Macon** | 0.62 | 0.66 | 0.40 | 1.69 |
| 27 | **Alabama** | **Bullock** | 0.64 | 0.64 | 0.40 | 1.68 |
| 28 | **Louisiana** | **Assumption** | 0.53 | 0.63 | 0.52 | 1.68 |
| 29 | Mississippi | Sunflower | 0.64 | 0.84 | 0.19 | 1.68 |
| 30 | **Georgia** | **Atkinson** | 0.78 | 0.59 | 0.31 | 1.68 |
| 31 | **Oregon** | **Josephine** | 0.33 | 0.53 | 0.81 | 1.66 |
| 32 | South Dakota | Ziebach | 0.91 | 0.68 | 0.06 | 1.66 |
| 33 | **Georgia** | **Baker** | 0.61 | 0.61 | 0.44 | 1.66 |
| 34 | South Dakota | Corson | 0.85 | 0.74 | 0.06 | 1.65 |
| 35 | **Georgia** | **Calhoun** | 0.70 | 0.55 | 0.40 | 1.65 |
| 36 | Mississippi | Quitman | 0.75 | 0.71 | 0.19 | 1.65 |
| 37 | **Mississippi** | **Leake** | 0.68 | 0.73 | 0.24 | 1.65 |
| 38 | **Mississippi** | **Claiborne** | 0.78 | 0.66 | 0.21 | 1.65 |
| 39 | **Florida** | **Gadsden** | 0.58 | 0.71 | 0.36 | 1.65 |
| 40 | Kentucky | Wolfe | 0.64 | 0.81 | 0.19 | 1.65 |
| 41 | South Dakota | Dewey | 0.78 | 0.81 | 0.06 | 1.64 |
| 42 | **Alabama** | **Hale** | 0.61 | 0.77 | 0.26 | 1.64 |
| 43 | **Mississippi** | **Scott** | 0.66 | 0.75 | 0.23 | 1.64 |
| 44 | **Georgia** | **Macon** | 0.61 | 0.68 | 0.35 | 1.64 |
| 45 | **Georgia** | **Colquitt** | 0.67 | 0.60 | 0.37 | 1.64 |
| 46 | **South Carolina** | **Allendale** | 0.71 | 0.63 | 0.29 | 1.63 |
| 47 | **Georgia** | **Crisp** | 0.64 | 0.66 | 0.33 | 1.63 |
| 48 | **Georgia** | **Early** | 0.60 | 0.67 | 0.36 | 1.63 |
| 49 | Mississippi | Pike | 0.56 | 0.86 | 0.21 | 1.63 |
| 50 | **Mississippi** | **Panola** | 0.56 | 0.83 | 0.23 | 1.62 |

|  | State | County | Adaptive capacity | Sensitivity | exposure | vulnerability |
| --- | --- | --- | --- | --- | --- | --- |
| 51 | Mississippi | Issaquena | 0.80 | 0.63 | 0.19 | 1.62 |
| 52 | **South Carolina** | **Williamsburg** | 0.60 | 0.80 | 0.22 | 1.62 |
| 53 | **Georgia** | **Mitchell** | 0.61 | 0.59 | 0.41 | 1.61 |
| 54 | **Alabama** | **Sumter** | 0.64 | 0.70 | 0.27 | 1.61 |
| 55 | **Mississippi** | **Kemper** | 0.63 | 0.71 | 0.27 | 1.61 |
| 56 | **Georgia** | **Warren** | 0.67 | 0.63 | 0.30 | 1.61 |
| 57 | Arkansas | Phillips | 0.57 | 0.82 | 0.22 | 1.60 |
| 58 | Florida | Hendry | 0.82 | 0.62 | 0.16 | 1.60 |
| 59 | **Georgia** | **Clinch** | 0.67 | 0.65 | 0.28 | 1.59 |
| 60 | **Alabama** | **Marengo** | 0.52 | 0.79 | 0.28 | 1.59 |
| 61 | Mississippi | Adams | 0.62 | 0.77 | 0.21 | 1.59 |
| 62 | **Georgia** | **Stewart** | 0.81 | 0.41 | 0.36 | 1.59 |
| 63 | **Georgia** | **Jenkins** | 0.61 | 0.67 | 0.31 | 1.59 |
| 64 | **Georgia** | **Grady** | 0.57 | 0.58 | 0.44 | 1.59 |
| 65 | **Mississippi** | **Neshoba** | 0.57 | 0.77 | 0.25 | 1.59 |
| 66 | **Alabama** | **Perry** | 0.51 | 0.80 | 0.28 | 1.58 |
| 67 | Texas | Presidio | 1.00 | 0.56 | 0.02 | 1.58 |
| 68 | **Georgia** | **Dooly** | 0.62 | 0.62 | 0.34 | 1.58 |
| 69 | **California** | **Trinity** | 0.33 | 0.38 | 0.86 | 1.57 |
| 70 | South Dakota | Buffalo | 0.90 | 0.61 | 0.06 | 1.57 |
| 71 | **Oklahoma** | **McIntosh** | 0.51 | 0.79 | 0.27 | 1.57 |
| 72 | **Kentucky** | **Breathitt** | 0.59 | 0.73 | 0.25 | 1.57 |
| 73 | **Georgia** | **Taliaferro** | 0.64 | 0.63 | 0.30 | 1.57 |
| 74 | **Georgia** | **Taylor** | 0.61 | 0.60 | 0.36 | 1.57 |
| 75 | **Alabama** | **Russell** | 0.51 | 0.66 | 0.41 | 1.57 |
| 76 | **Georgia** | **Sumter** | 0.55 | 0.64 | 0.37 | 1.57 |
| 77 | **Oklahoma** | **Cherokee** | 0.52 | 0.71 | 0.35 | 1.57 |
| 78 | Kentucky | Harlan | 0.52 | 0.85 | 0.20 | 1.56 |
| 79 | **Alabama** | **Monroe** | 0.54 | 0.75 | 0.27 | 1.56 |
| 80 | **Alabama** | **Clarke** | 0.51 | 0.78 | 0.26 | 1.56 |
| 81 | West Virginia | Boone | 0.40 | 0.92 | 0.25 | 1.56 |
| 82 | Mississippi | Leflore | 0.56 | 0.81 | 0.19 | 1.56 |
| 83 | **Georgia** | **Turner** | 0.61 | 0.61 | 0.33 | 1.55 |
| 84 | Oklahoma | McCurtain | 0.52 | 0.78 | 0.25 | 1.55 |
| 85 | **Alabama** | **Conecuh** | 0.57 | 0.70 | 0.29 | 1.55 |
| 86 | Mississippi | Attala | 0.56 | 0.76 | 0.23 | 1.55 |
| 87 | **Georgia** | **Decatur** | 0.48 | 0.66 | 0.41 | 1.55 |
| 88 | **Mississippi** | **Winston** | 0.55 | 0.74 | 0.26 | 1.55 |
| 89 | Oklahoma | Choctaw | 0.52 | 0.80 | 0.22 | 1.54 |
| 90 | **Texas** | **Jefferson** | 0.45 | 0.57 | 0.52 | 1.54 |
| 91 | Arizona | Apache | 0.71 | 0.79 | 0.04 | 1.54 |
| 92 | **Georgia** | **Jefferson** | 0.58 | 0.65 | 0.32 | 1.54 |
| 93 | **Oklahoma** | **Sequoyah** | 0.50 | 0.71 | 0.33 | 1.54 |
| 94 | **Georgia** | **Johnson** | 0.62 | 0.60 | 0.33 | 1.54 |
| 95 | **Oklahoma** | **Delaware** | 0.50 | 0.75 | 0.29 | 1.54 |
| 96 | Mississippi | Walthall | 0.53 | 0.80 | 0.21 | 1.54 |
| 97 | **Georgia** | **Treutlen** | 0.63 | 0.59 | 0.32 | 1.54 |
| 98 | **Louisiana** | **Iberia** | 0.49 | 0.66 | 0.39 | 1.54 |
| 99 | Mississippi | Tallahatchie | 0.66 | 0.68 | 0.20 | 1.54 |
| 100 | **Georgia** | **Laurens** | 0.50 | 0.72 | 0.32 | 1.54 |

Supplementary Table S10. The 100 least vulnerable counties using the multiplicative method. Counties identified as the 100 least vulnerable counties from both multiplicative and additive methods are in bold.

|  | State | County | Adaptive capacity | Sensitivity | exposure | vulnerability |
| --- | --- | --- | --- | --- | --- | --- |
| 1 | **Virginia** | **Falls Church** | 0.00 | 0.09 | 0.07 | 0.0000 |
| 2 | **California** | **San Francisco** | 0.12 | 0.00 | 0.15 | 0.0000 |
| 3 | Maine | Aroostook | 0.29 | 0.67 | 0.00 | 0.0000 |
| 4 | **Utah** | **Summit** | 0.14 | 0.00 | 0.08 | 0.0001 |
| 5 | **Virginia** | **Arlington** | 0.07 | 0.02 | 0.07 | 0.0001 |
| 6 | **Vermont** | **Chittenden** | 0.16 | 0.06 | 0.01 | 0.0001 |
| 7 | **Rhode Island** | **Bristol** | 0.03 | 0.27 | 0.02 | 0.0002 |
| 8 | **Colorado** | **Douglas** | 0.06 | 0.05 | 0.06 | 0.0002 |
| 9 | Massachusetts | Barnstable | 0.03 | 0.43 | 0.02 | 0.0003 |
| 10 | **Montana** | **Gallatin** | 0.17 | 0.01 | 0.14 | 0.0003 |
| 11 | **Vermont** | **Grand Isle** | 0.14 | 0.17 | 0.01 | 0.0003 |
| 12 | **Maine** | **Cumberland** | 0.14 | 0.26 | 0.01 | 0.0003 |
| 13 | **New Jersey** | **Hunterdon** | 0.06 | 0.11 | 0.05 | 0.0003 |
| 14 | **New Jersey** | **Morris** | 0.10 | 0.08 | 0.04 | 0.0003 |
| 15 | **New York** | **Nassau** | 0.04 | 0.20 | 0.04 | 0.0003 |
| 16 | **New Hampshire** | **Rockingham** | 0.09 | 0.30 | 0.01 | 0.0004 |
| 17 | **Vermont** | **Lamoille** | 0.19 | 0.16 | 0.01 | 0.0004 |
| 18 | **Vermont** | **Addison** | 0.17 | 0.15 | 0.02 | 0.0004 |
| 19 | **New Jersey** | **Bergen** | 0.13 | 0.08 | 0.04 | 0.0004 |
| 20 | **Massachusetts** | **Middlesex** | 0.09 | 0.25 | 0.02 | 0.0004 |
| 21 | **New Mexico** | **Los Alamos** | 0.05 | 0.12 | 0.07 | 0.0004 |
| 22 | **Vermont** | **Washington** | 0.17 | 0.19 | 0.01 | 0.0004 |
| 23 | **Colorado** | **Gilpin** | 0.05 | 0.16 | 0.06 | 0.0005 |
| 24 | **New Jersey** | **Somerset** | 0.12 | 0.08 | 0.05 | 0.0005 |
| 25 | **Colorado** | **Boulder** | 0.17 | 0.04 | 0.07 | 0.0005 |
| 26 | Maine | Sagadahoc | 0.18 | 0.42 | 0.01 | 0.0005 |
| 27 | **Massachusetts** | **Norfolk** | 0.09 | 0.30 | 0.02 | 0.0005 |
| 28 | Massachusetts | Dukes | 0.05 | 0.44 | 0.02 | 0.0005 |
| 29 | Maine | Knox | 0.21 | 0.41 | 0.01 | 0.0005 |
| 30 | Massachusetts | Nantucket | 0.08 | 0.38 | 0.02 | 0.0006 |
| 31 | Maine | Lincoln | 0.23 | 0.40 | 0.01 | 0.0006 |
| 32 | **Rhode Island** | **Washington** | 0.08 | 0.31 | 0.03 | 0.0006 |
| 33 | **New York** | **Putnam** | 0.10 | 0.19 | 0.04 | 0.0006 |
| 34 | Vermont | Caledonia | 0.19 | 0.30 | 0.01 | 0.0006 |
| 35 | **Vermont** | **Windham** | 0.20 | 0.18 | 0.02 | 0.0007 |
| 36 | Maine | Hancock | 0.18 | 0.44 | 0.01 | 0.0007 |
| 37 | **Colorado** | **Summit** | 0.17 | 0.08 | 0.05 | 0.0007 |
| 38 | **Rhode Island** | **Newport** | 0.10 | 0.30 | 0.02 | 0.0007 |
| 39 | **Vermont** | **Windsor** | 0.17 | 0.27 | 0.02 | 0.0007 |
| 40 | Maine | Franklin | 0.23 | 0.48 | 0.01 | 0.0007 |
| 41 | New Hampshire | Merrimack | 0.15 | 0.35 | 0.01 | 0.0007 |
| 42 | **Wisconsin** | **Ozaukee** | 0.07 | 0.19 | 0.06 | 0.0007 |
| 43 | Vermont | Franklin | 0.19 | 0.31 | 0.01 | 0.0007 |
| 44 | New Hampshire | Grafton | 0.17 | 0.33 | 0.01 | 0.0007 |
| 45 | **Minnesota** | **Carver** | 0.09 | 0.12 | 0.07 | 0.0007 |
| 46 | **New York** | **Suffolk** | 0.09 | 0.23 | 0.03 | 0.0007 |
| 47 | **Colorado** | **Broomfield** | 0.08 | 0.14 | 0.06 | 0.0008 |
| 48 | Maine | Piscataquis | 0.29 | 0.61 | 0.00 | 0.0008 |
| 49 | Vermont | Orange | 0.20 | 0.30 | 0.01 | 0.0008 |
| 50 | Maine | York | 0.17 | 0.39 | 0.01 | 0.0008 |
|  | State | County | Adaptive capacity | Sensitivity | exposure | vulnerability |
| 51 | **Virginia** | **Loudoun** | 0.12 | 0.08 | 0.08 | 0.0008 |
| 52 | Maine | Androscoggin | 0.25 | 0.50 | 0.01 | 0.0009 |
| 53 | **Colorado** | **Pitkin** | 0.18 | 0.12 | 0.04 | 0.0009 |
| 54 | **New York** | **Saratoga** | 0.13 | 0.30 | 0.02 | 0.0009 |
| 55 | New Hampshire | Carroll | 0.17 | 0.44 | 0.01 | 0.0009 |
| 56 | **Massachusetts** | **Hampshire** | 0.15 | 0.29 | 0.02 | 0.0009 |
| 57 | Vermont | Orleans | 0.23 | 0.37 | 0.01 | 0.0009 |
| 58 | **Connecticut** | **Tolland** | 0.12 | 0.31 | 0.02 | 0.0009 |
| 59 | Maine | Kennebec | 0.23 | 0.50 | 0.01 | 0.0009 |
| 60 | Vermont | Essex | 0.27 | 0.37 | 0.01 | 0.0009 |
| 61 | **Minnesota** | **Washington** | 0.09 | 0.15 | 0.07 | 0.0009 |
| 62 | **Virginia** | **Fairfax** | 0.15 | 0.08 | 0.08 | 0.0010 |
| 63 | New Hampshire | Strafford | 0.19 | 0.39 | 0.01 | 0.0010 |
| 64 | **New Jersey** | **Sussex** | 0.11 | 0.20 | 0.04 | 0.0010 |
| 65 | Connecticut | Middlesex | 0.10 | 0.35 | 0.03 | 0.0010 |
| 66 | Vermont | Rutland | 0.16 | 0.33 | 0.02 | 0.0010 |
| 67 | Massachusetts | Plymouth | 0.13 | 0.41 | 0.02 | 0.0010 |
| 68 | **Colorado** | **San Miguel** | 0.16 | 0.15 | 0.04 | 0.0010 |
| 69 | New Hampshire | Belknap | 0.20 | 0.43 | 0.01 | 0.0010 |
| 70 | **New York** | **New York** | 0.32 | 0.09 | 0.04 | 0.0010 |
| 71 | **Wisconsin** | **Waukesha** | 0.07 | 0.21 | 0.07 | 0.0011 |
| 72 | New Hampshire | Hillsborough | 0.18 | 0.38 | 0.02 | 0.0011 |
| 73 | Maine | Washington | 0.39 | 0.66 | 0.00 | 0.0011 |
| 74 | **Colorado** | **Jefferson** | 0.16 | 0.13 | 0.05 | 0.0011 |
| 75 | New York | Essex | 0.18 | 0.37 | 0.02 | 0.0011 |
| 76 | Rhode Island | Kent | 0.11 | 0.44 | 0.02 | 0.0011 |
| 77 | **North Dakota** | **Dickey** | 0.06 | 0.30 | 0.06 | 0.0011 |
| 78 | Maine | Somerset | 0.35 | 0.61 | 0.01 | 0.0011 |
| 79 | **Minnesota** | **Scott** | 0.13 | 0.13 | 0.07 | 0.0012 |
| 80 | **Minnesota** | **Hennepin** | 0.16 | 0.10 | 0.07 | 0.0012 |
| 81 | Vermont | Bennington | 0.22 | 0.26 | 0.02 | 0.0012 |
| 82 | Massachusetts | Essex | 0.19 | 0.41 | 0.02 | 0.0012 |
| 83 | **Pennsylvania** | **Montgomery** | 0.10 | 0.23 | 0.05 | 0.0012 |
| 84 | **Maryland** | **Howard** | 0.12 | 0.15 | 0.07 | 0.0012 |
| 85 | New Hampshire | Coos | 0.27 | 0.51 | 0.01 | 0.0013 |
| 86 | New York | Warren | 0.17 | 0.39 | 0.02 | 0.0013 |
| 87 | Maine | Oxford | 0.28 | 0.57 | 0.01 | 0.0013 |
| 88 | **Colorado** | **Gunnison** | 0.20 | 0.16 | 0.04 | 0.0013 |
| 89 | Massachusetts | Suffolk | 0.23 | 0.34 | 0.02 | 0.0013 |
| 90 | **New York** | **Tompkins** | 0.22 | 0.17 | 0.04 | 0.0013 |
| 91 | New Hampshire | Cheshire | 0.19 | 0.41 | 0.02 | 0.0014 |
| 92 | **Colorado** | **Elbert** | 0.11 | 0.23 | 0.06 | 0.0014 |
| 93 | New York | Albany | 0.19 | 0.29 | 0.03 | 0.0014 |
| 94 | Massachusetts | Franklin | 0.16 | 0.45 | 0.02 | 0.0014 |
| 95 | Connecticut | Litchfield | 0.12 | 0.39 | 0.03 | 0.0014 |
| 96 | North Dakota | Ransom | 0.07 | 0.33 | 0.07 | 0.0015 |
| 97 | **Colorado** | **Clear Creek** | 0.13 | 0.22 | 0.05 | 0.0015 |
| 98 | **Colorado** | **Ouray** | 0.15 | 0.25 | 0.04 | 0.0015 |
| 99 | **New Jersey** | **Monmouth** | 0.11 | 0.22 | 0.06 | 0.0015 |
| 100 | **New York** | **Richmond** | 0.09 | 0.31 | 0.05 | 0.0015 |

Supplementary Table S11. The 100 least vulnerable counties using the additive method. Counties identified as the 100 least vulnerable counties from both multiplicative and additive methods are in bold.

|  | State | County | Adaptive capacity | Sensitivity | exposure | vulnerability |
| --- | --- | --- | --- | --- | --- | --- |
| 1 | **Virginia** | **Falls Church** | 0.00 | 0.09 | 0.07 | 0.16 |
| 2 | **Virginia** | **Arlington** | 0.07 | 0.02 | 0.07 | 0.16 |
| 3 | **Colorado** | **Douglas** | 0.06 | 0.05 | 0.06 | 0.18 |
| 4 | **New Jersey** | **Morris** | 0.10 | 0.08 | 0.04 | 0.22 |
| 5 | **New Jersey** | **Hunterdon** | 0.06 | 0.11 | 0.05 | 0.22 |
| 6 | **Utah** | **Summit** | 0.14 | 0.00 | 0.08 | 0.23 |
| 7 | **Vermont** | **Chittenden** | 0.16 | 0.06 | 0.01 | 0.24 |
| 8 | **New Mexico** | **Los Alamos** | 0.05 | 0.12 | 0.07 | 0.24 |
| 9 | **New Jersey** | **Somerset** | 0.12 | 0.08 | 0.05 | 0.25 |
| 10 | **New Jersey** | **Bergen** | 0.13 | 0.08 | 0.04 | 0.25 |
| 11 | **Colorado** | **Gilpin** | 0.05 | 0.16 | 0.06 | 0.26 |
| 12 | **California** | **San Francisco** | 0.12 | 0.00 | 0.15 | 0.27 |
| 13 | **Minnesota** | **Carver** | 0.09 | 0.12 | 0.07 | 0.28 |
| 14 | **New York** | **Nassau** | 0.04 | 0.20 | 0.04 | 0.28 |
| 15 | **Colorado** | **Boulder** | 0.17 | 0.04 | 0.07 | 0.28 |
| 16 | **Virginia** | **Loudoun** | 0.12 | 0.08 | 0.08 | 0.29 |
| 17 | **Colorado** | **Broomfield** | 0.08 | 0.14 | 0.06 | 0.29 |
| 18 | **Colorado** | **Summit** | 0.17 | 0.08 | 0.05 | 0.29 |
| 19 | **Minnesota** | **Washington** | 0.09 | 0.15 | 0.07 | 0.31 |
| 20 | **Virginia** | **Fairfax** | 0.15 | 0.08 | 0.08 | 0.31 |
| 21 | **Wisconsin** | **Ozaukee** | 0.07 | 0.19 | 0.06 | 0.32 |
| 22 | **New York** | **Putnam** | 0.10 | 0.19 | 0.04 | 0.32 |
| 23 | **Rhode Island** | **Bristol** | 0.03 | 0.27 | 0.02 | 0.32 |
| 24 | **Vermont** | **Grand Isle** | 0.14 | 0.17 | 0.01 | 0.33 |
| 25 | **Montana** | **Gallatin** | 0.17 | 0.01 | 0.14 | 0.33 |
| 26 | **Minnesota** | **Scott** | 0.13 | 0.13 | 0.07 | 0.33 |
| 27 | **Vermont** | **Addison** | 0.17 | 0.15 | 0.02 | 0.33 |
| 28 | **Colorado** | **Pitkin** | 0.18 | 0.12 | 0.04 | 0.34 |
| 29 | **Minnesota** | **Hennepin** | 0.16 | 0.10 | 0.07 | 0.34 |
| 30 | **Maryland** | **Howard** | 0.12 | 0.15 | 0.07 | 0.34 |
| 31 | **Colorado** | **Jefferson** | 0.16 | 0.13 | 0.05 | 0.34 |
| 32 | **Wisconsin** | **Waukesha** | 0.07 | 0.21 | 0.07 | 0.35 |
| 33 | **New Jersey** | **Sussex** | 0.11 | 0.20 | 0.04 | 0.35 |
| 34 | **Colorado** | **San Miguel** | 0.16 | 0.15 | 0.04 | 0.35 |
| 35 | **Massachusetts** | **Middlesex** | 0.09 | 0.25 | 0.02 | 0.36 |
| 36 | **New York** | **Suffolk** | 0.09 | 0.23 | 0.03 | 0.36 |
| 37 | **Vermont** | **Lamoille** | 0.19 | 0.16 | 0.01 | 0.36 |
| 38 | Colorado | Routt | 0.14 | 0.15 | 0.08 | 0.36 |
| 39 | Iowa | Dallas | 0.13 | 0.14 | 0.10 | 0.37 |
| 40 | **Vermont** | **Washington** | 0.17 | 0.19 | 0.01 | 0.37 |
| 41 | Virginia | Fairfax | 0.11 | 0.18 | 0.08 | 0.38 |
| 42 | Minnesota | Wright | 0.14 | 0.17 | 0.07 | 0.38 |
| 43 | Wisconsin | Dane | 0.14 | 0.16 | 0.08 | 0.38 |
| 44 | **Pennsylvania** | **Montgomery** | 0.10 | 0.23 | 0.05 | 0.38 |
| 45 | Indiana | Hamilton | 0.11 | 0.18 | 0.09 | 0.39 |
| 46 | Wisconsin | St. Croix | 0.11 | 0.22 | 0.07 | 0.39 |
| 47 | Wisconsin | La Crosse | 0.16 | 0.16 | 0.07 | 0.39 |
| 48 | California | San Mateo | 0.16 | 0.08 | 0.15 | 0.39 |
| 49 | **New Jersey** | **Monmouth** | 0.11 | 0.22 | 0.06 | 0.40 |
| 50 | **Colorado** | **Elbert** | 0.11 | 0.23 | 0.06 | 0.40 |

|  | State | County | Adaptive capacity | Sensitivity | exposure | vulnerability |
| --- | --- | --- | --- | --- | --- | --- |
| 51 | Illinois | DuPage | 0.11 | 0.20 | 0.09 | 0.40 |
| 52 | South Dakota | Lincoln | 0.13 | 0.19 | 0.08 | 0.40 |
| 53 | Pennsylvania | Chester | 0.13 | 0.21 | 0.06 | 0.40 |
| 54 | Minnesota | Dakota | 0.14 | 0.18 | 0.07 | 0.40 |
| 55 | **Vermont** | **Windham** | 0.20 | 0.18 | 0.02 | 0.40 |
| 56 | **Colorado** | **Gunnison** | 0.20 | 0.16 | 0.04 | 0.40 |
| 57 | Colorado | Larimer | 0.19 | 0.14 | 0.08 | 0.40 |
| 58 | **Colorado** | **Clear Creek** | 0.13 | 0.22 | 0.05 | 0.40 |
| 59 | **New Hampshire** | **Rockingham** | 0.09 | 0.30 | 0.01 | 0.41 |
| 60 | **Maine** | **Cumberland** | 0.14 | 0.26 | 0.01 | 0.41 |
| 61 | Minnesota | Nicollet | 0.20 | 0.14 | 0.07 | 0.41 |
| 62 | Wisconsin | Washington | 0.11 | 0.24 | 0.06 | 0.41 |
| 63 | **Massachusetts** | **Norfolk** | 0.09 | 0.30 | 0.02 | 0.41 |
| 64 | Minnesota | Chisago | 0.11 | 0.23 | 0.07 | 0.41 |
| 65 | Virginia | Alexandria | 0.19 | 0.15 | 0.08 | 0.41 |
| 66 | Utah | Morgan | 0.14 | 0.20 | 0.08 | 0.42 |
| 67 | **Rhode Island** | **Washington** | 0.08 | 0.31 | 0.03 | 0.42 |
| 68 | California | Marin | 0.13 | 0.09 | 0.20 | 0.42 |
| 69 | Wisconsin | Calumet | 0.13 | 0.23 | 0.06 | 0.42 |
| 70 | **Rhode Island** | **Newport** | 0.10 | 0.30 | 0.02 | 0.42 |
| 71 | Ohio | Delaware | 0.11 | 0.23 | 0.08 | 0.42 |
| 72 | **North Dakota** | **Dickey** | 0.06 | 0.30 | 0.06 | 0.42 |
| 73 | **New York** | **Tompkins** | 0.22 | 0.17 | 0.04 | 0.43 |
| 74 | Virginia | Poquoson | 0.07 | 0.26 | 0.10 | 0.43 |
| 75 | Minnesota | Olmsted | 0.17 | 0.18 | 0.07 | 0.43 |
| 76 | Washington | King | 0.16 | 0.15 | 0.11 | 0.43 |
| 77 | Wyoming | Teton | 0.17 | 0.12 | 0.14 | 0.43 |
| 78 | Colorado | Eagle | 0.28 | 0.10 | 0.06 | 0.44 |
| 79 | Colorado | Park | 0.14 | 0.23 | 0.06 | 0.44 |
| 80 | California | Santa Clara | 0.20 | 0.06 | 0.19 | 0.44 |
| 81 | **Colorado** | **Ouray** | 0.15 | 0.25 | 0.04 | 0.44 |
| 82 | **New York** | **New York** | 0.32 | 0.09 | 0.04 | 0.44 |
| 83 | Minnesota | Sherburne | 0.14 | 0.23 | 0.07 | 0.45 |
| 84 | **Vermont** | **Windsor** | 0.17 | 0.27 | 0.02 | 0.45 |
| 85 | Minnesota | Dodge | 0.15 | 0.23 | 0.07 | 0.45 |
| 86 | Iowa | Johnson | 0.22 | 0.13 | 0.10 | 0.45 |
| 87 | Minnesota | Lincoln | 0.09 | 0.30 | 0.07 | 0.45 |
| 88 | Minnesota | Houston | 0.11 | 0.26 | 0.08 | 0.45 |
| 89 | **Massachusetts** | **Hampshire** | 0.15 | 0.29 | 0.02 | 0.45 |
| 90 | **New York** | **Saratoga** | 0.13 | 0.30 | 0.02 | 0.45 |
| 91 | Minnesota | Le Sueur | 0.17 | 0.21 | 0.07 | 0.46 |
| 92 | Iowa | Winneshiek | 0.11 | 0.26 | 0.08 | 0.46 |
| 93 | Minnesota | Douglas | 0.12 | 0.26 | 0.07 | 0.46 |
| 94 | Maryland | Montgomery | 0.22 | 0.16 | 0.07 | 0.46 |
| 95 | Iowa | Bremer | 0.10 | 0.27 | 0.09 | 0.46 |
| 96 | **Connecticut** | **Tolland** | 0.12 | 0.31 | 0.02 | 0.46 |
| 97 | **New York** | **Richmond** | 0.09 | 0.31 | 0.05 | 0.46 |
| 98 | Minnesota | Winona | 0.16 | 0.23 | 0.07 | 0.46 |
| 99 | New York | Westchester | 0.22 | 0.20 | 0.04 | 0.46 |
| 100 | South Dakota | Clay | 0.26 | 0.12 | 0.08 | 0.47 |

Supplementary Table S12. Descriptive summary of daily average fire-PM_2.5_ (μg/m^3^) by decile, and the number of counties by daily average fire-PM_2.5_ and the decile.

|  |  | Adaptive capacity (decile) | | | | | | | | | | |
| --- | --- | --- | --- | --- | --- | --- | --- | --- | --- | --- | --- | --- |
|  |  | 1st | 2nd | 3rd | 4th | 5th | 6th | 7th | 8th | 9th | 10th | Average |
| PM_2.5_  (μg/m^3^) | Mean | 0.54 | 0.59 | 0.64 | 0.73 | 0.88 | 1.00 | 1.07 | 1.13 | 1.22 | 1.14 | 0.89 |
|  | Max | 2.70 | 2.73 | 2.69 | 3.21 | 4.37 | 5.16 | 3.13 | 5.96 | 2.82 | 3.14 | 5.96 |
|  | Min | 0.11 | 0.10 | 0.11 | 0.11 | 0.07 | 0.10 | 0.09 | 0.21 | 0.25 | 0.17 | 0.07 |
|  | SD | 0.30 | 0.31 | 0.37 | 0.38 | 0.57 | 0.58 | 0.51 | 0.58 | 0.55 | 0.65 | 0.55 |
| Number of counties | 0.00-0.15  (μg/m^3^) | 9  (2.9) | 8  (2.6) | 4  (1.3) | 3  (1.0) | 3  (1.0) | 1  (0.3) | 1  (0.3) | 0  (0.0) | 0  (0.0) | 0  (0.0) | 29  (0.9) |
|  | 0.15-0.75  (μg/m^3^) | 268  (86.2) | 243  (78.1) | 228  (73.3) | 182  (58.7) | 148  (47.6) | 102  (32.8) | 84  (27.1) | 72  (23.2) | 68  (21.9) | 106  (34.1) | 1501  (48.3) |
|  | 0.75-1.50  (μg/m^3^) | 30  (9.6) | 51  (16.4) | 66  (21.2) | 115  (37.1) | 129  (41.5) | 173  (55.6) | 162  (52.3) | 179  (57.6) | 146  (46.9) | 115  (37.0) | 1166  (37.5) |
|  | >1.50  (μg/m^3^) | 4  (1.3) | 9  (2.9) | 13  (4.2) | 10  (3.2) | 31  (10.0) | 35  (11.3) | 63  (20.3) | 60  (19.3) | 97  (31.2) | 90  (28.9) | 412  (13.3) |
|  |  | Sensitivity (decile) | | | | | | | | | | |
|  |  | 1st | 2nd | 3rd | 4th | 5th | 6th | 7th | 8th | 9th | 10th | Average |
| PM_2.5_  (μg/m^3^) | Mean | 0.65 | 0.67 | 0.66 | 0.71 | 0.78 | 0.84 | 0.99 | 1.12 | 1.25 | 1.27 | 0.89 |
|  | Max | 3.26 | 5.96 | 2.80 | 5.16 | 4.37 | 3.49 | 3.90 | 4.84 | 3.14 | 2.58 | 5.96 |
|  | Min | 0.12 | 0.13 | 0.15 | 0.11 | 0.10 | 0.12 | 0.11 | 0.11 | 0.09 | 0.07 | 0.07 |
|  | SD | 0.46 | 0.56 | 0.40 | 0.44 | 0.51 | 0.50 | 0.53 | 0.60 | 0.49 | 0.47 | 0.55 |
| Number of counties | 0.00-0.15  (μg/m^3^) | 4  (1.3) | 5  (1.6) | 1  (0.3) | 5  (1.6) | 3  (1.0) | 2  (0.6) | 4  (1.3) | 1  (0.3) | 2  (0.6) | 2  (0.6) | 29  (0.9) |
|  | 0.15-0.75  (μg/m^3^) | 240  (77.2) | 239  (76.8) | 229  (73.6) | 194  (62.6) | 178  (57.2) | 157  (50.5) | 105  (33.9) | 89  (28.6) | 32  (10.3) | 38  (12.2) | 1501  (48.3) |
|  | 0.75-1.50  (μg/m^3^) | 50  (16.1) | 47  (15.1) | 67  (21.5) | 97  (31.3) | 109  (35.0) | 121  (38.9) | 146  (47.1) | 146  (46.9) | 203  (65.3) | 180  (57.9) | 1166  (37.5) |
|  | >1.50  (μg/m^3^) | 17  (5.5) | 20  (6.4) | 14  (4.5) | 14  (4.5) | 21  (6.8) | 31  (10.0) | 55  (17.7) | 75  (24.1) | 74  (23.8) | 91  (29.3) | 412  (13.3) |
|  |  | Community health vulnerability index (decile) | | | | | | | | | | |
|  |  | 1st | 2nd | 3rd | 4th | 5th | 6th | 7th | 8th | 9th | 10th | Average |
| PM_2.5_  (μg/m^3^) | Mean | 0.37 | 0.46 | 0.5 | 0.59 | 0.67 | 0.85 | 1.04 | 1.2 | 1.45 | 1.82 | 0.89 |
|  | Max | 1.28 | 1.07 | 1.16 | 2.7 | 2.39 | 2.69 | 2.73 | 3.21 | 3.26 | 5.96 | 5.96 |
|  | Min | 0.07 | 0.17 | 0.22 | 0.21 | 0.19 | 0.25 | 0.33 | 0.4 | 0.47 | 0.59 | 0.07 |
|  | SD | 0.18 | 0.14 | 0.15 | 0.24 | 0.24 | 0.29 | 0.34 | 0.37 | 0.37 | 0.58 | 0.55 |
| Number of counties | 0.00-0.15  (μg/m^3^) | 29  (9.3) | 0  (0.0) | 0  (0.0) | 0  (0.0) | 0  (0.0) | 0  (0.0) | 0  (0.0) | 0  (0.0) | 0  (0.0) | 0  (0.0) | 29  (0.9) |
|  | 0.15-0.75  (μg/m^3^) | 275  (88.4) | 298  (95.8) | 294  (94.5) | 261  (84.2) | 211  (67.8) | 115  (37.0) | 33  (10.6) | 12  (3.9) | 1  (0.3) | 1  (0.3) | 1501  (48.3) |
|  | 0.75-1.50  (μg/m^3^) | 7  (2.3) | 13  (4.2) | 17  (5.5) | 47  (15.2) | 96  (30.9) | 184  (59.2) | 254  (81.9) | 255  (82.0) | 204  (65.6) | 89  (28.6) | 1166  (37.5) |
|  | >1.50  (μg/m^3^) | 0  (0.0) | 0  (0.0) | 0  (0.0) | 2  (0.6) | 4  (1.3) | 12  (3.9) | 23  (7.4) | 44  (14.1) | 106  (34.1) | 221  (71.1) | 412  (13.3) |

Supplementary Table S13. Number of moderate and at or above unhealthy air quality days for sensitive groups per year based on all-sources PM_2.5_.

|  | Adaptive capacity sub-index (decile) | | | | | | | | | | |
| --- | --- | --- | --- | --- | --- | --- | --- | --- | --- | --- | --- |
|  | 1st | 2nd | 3rd | 4th | 5th | 6th | 7th | 8th | 9th | 10th | Average |
| Moderate air quality (12-35μg/m^3^) | 51.71 | 47.91 | 44.52 | 48.23 | 48.09 | 49.58 | 45.98 | 44.92 | 44.16 | 38.60 | 46.37 |
| At or above unhealthy air quality for sensitive groups (>35μg/m^3^) | 0.98 | 0.70 | 0.75 | 0.77 | 1.07 | 1.08 | 1.19 | 1.05 | 1.14 | 1.16 | 0.99 |
|  | Sensitivity sub-index (decile) | | | | | | | | | | |
|  | 1st | 2nd | 3rd | 4th | 5th | 6th | 7th | 8th | 9th | 10th | Average |
| Moderate air quality (12-35μg/m^3^) | 45.14 | 37.04 | 36.37 | 42.39 | 41.35 | 48.36 | 52.84 | 54.60 | 54.03 | 51.57 | 46.37 |
| At or above unhealthy air quality for sensitive groups (>35μg/m^3^) | 1.47 | 0.91 | 0.69 | 0.71 | 0.71 | 0.92 | 1.06 | 1.16 | 1.19 | 1.08 | 0.99 |
|  | Community health vulnerability index (decile) | | | | | | | | | | |
|  | 1st | 2nd | 3rd | 4th | 5th | 6th | 7th | 8th | 9th | 10th | Average |
| Moderate air quality (12-35μg/m^3^) | 44.93 | 36.36 | 41.23 | 44.44 | 42.89 | 45.13 | 46.48 | 48.65 | 55.21 | 58.37 | 46.37 |
| At or above unhealthy air quality for sensitive groups (>35μg/m^3^) | 1.16 | 0.46 | 0.54 | 0.63 | 0.68 | 0.66 | 0.90 | 1.08 | 1.44 | 2.35 | 0.99 |

Supplementary Table S14. Number of moderate and at or above unhealthy air quality days for sensitive groups per year based on all-sources PM_2.5_ without wildfires and prescribed fires.

|  | Adaptive capacity sub-index (decile) | | | | | | | | | | |
| --- | --- | --- | --- | --- | --- | --- | --- | --- | --- | --- | --- |
|  | 1st | 2nd | 3rd | 4th | 5th | 6th | 7th | 8th | 9th | 10th | Average |
| Moderate air quality (12-35μg/m^3^) | 42.14 | 37.31 | 34.03 | 35.08 | 33.53 | 31.63 | 26.92 | 24.38 | 21.93 | 18.63 | 30.56 |
| At or above unhealthy air quality for sensitive groups (>35μg/m^3^) | 0.52 | 0.25 | 0.19 | 0.19 | 0.17 | 0.23 | 0.28 | 0.09 | 0.08 | 0.11 | 0.21 |
|  | Sensitivity sub-index (decile) | | | | | | | | | | |
|  | 1st | 2nd | 3rd | 4th | 5th | 6th | 7th | 8th | 9th | 10th | Average |
| Moderate air quality (12-35μg/m^3^) | 35.92 | 27.50 | 26.12 | 30.19 | 28.18 | 33.86 | 34.13 | 33.52 | 29.66 | 26.52 | 30.56 |
| At or above unhealthy air quality for sensitive groups (>35μg/m^3^) | 0.78 | 0.26 | 0.18 | 0.18 | 0.11 | 0.16 | 0.19 | 0.13 | 0.08 | 0.05 | 0.21 |
|  | Community health vulnerability index (decile) | | | | | | | | | | |
|  | 1st | 2nd | 3rd | 4th | 5th | 6th | 7th | 8th | 9th | 10th | Average |
| Moderate air quality (12-35μg/m^3^) | 38.99 | 28.90 | 32.94 | 34.73 | 31.9 | 29.69 | 27.52 | 26.70 | 28.02 | 26.19 | 30.56 |
| At or above unhealthy air quality for sensitive groups (>35μg/m^3^) | 0.83 | 0.23 | 0.26 | 0.22 | 0.20 | 0.08 | 0.08 | 0.04 | 0.09 | 0.09 | 0.21 |

Supplementary Table S15. Increases in the number of moderate and at or above unhealthy air quality days for sensitive groups per year due to wildland fires.

|  | Adaptive capacity sub-index (decile) | | | | | | | | | | |
| --- | --- | --- | --- | --- | --- | --- | --- | --- | --- | --- | --- |
|  | 1st | 2nd | 3rd | 4th | 5th | 6th | 7th | 8th | 9th | 10th | Average |
| Moderate air quality (12-35μg/m^3^) | 9.57 | 10.6 | 10.49 | 13.15 | 14.56 | 17.95 | 19.06 | 20.54 | 22.23 | 19.97 | 15.81 |
| At or above unhealthy air quality for sensitive groups (>35μg/m^3^) | 0.46 | 0.45 | 0.56 | 0.58 | 0.90 | 0.85 | 0.91 | 0.96 | 1.06 | 1.05 | 0.78 |
|  | Sensitivity sub-index (decile) | | | | | | | | | | |
|  | 1st | 2nd | 3rd | 4th | 5th | 6th | 7th | 8th | 9th | 10th | Average |
| Moderate air quality (12-35μg/m^3^) | 9.22 | 9.54 | 10.25 | 12.20 | 13.17 | 14.50 | 18.71 | 21.08 | 24.37 | 25.05 | 15.81 |
| At or above unhealthy air quality for sensitive groups (>35μg/m^3^) | 0.69 | 0.65 | 0.51 | 0.53 | 0.60 | 0.76 | 0.87 | 1.03 | 1.11 | 1.03 | 0.78 |
|  | Community health vulnerability index (decile) | | | | | | | | | | |
|  | 1st | 2nd | 3rd | 4th | 5th | 6th | 7th | 8th | 9th | 10th | Average |
| Moderate air quality (12-35μg/m^3^) | 5.94 | 7.46 | 8.29 | 9.71 | 10.99 | 15.44 | 18.96 | 21.95 | 27.19 | 32.18 | 15.81 |
| At or above unhealthy air quality for sensitive groups (>35μg/m^3^) | 0.33 | 0.23 | 0.28 | 0.41 | 0.48 | 0.58 | 0.82 | 1.04 | 1.35 | 2.26 | 0.78 |

Supplementary Table S16. Population size by daily average fire-PM_2.5_ concentration threshold and indices (adaptive capacity, sensitivity, and CHVI)

|  | Adaptive capacity sub-index (decile) | | | | | | | | | | |
| --- | --- | --- | --- | --- | --- | --- | --- | --- | --- | --- | --- |
|  | 1st | 2nd | 3rd | 4th | 5th | 6th | 7th | 8th | 9th | 10th | Total (100) |
| 0.00-0.15 | 1,191,638  (2.7) | 417,947  (1.3) | 212,837  (0.5) | 145,543  (0.4) | 142,195  (0.4) | 50,520  (0.2) | 31,491  (0.1) | 0  (0.0) | 0  (0.0) | 0  (0.0) | 2,192,171  (0.7) |
| 0.15-0.75 | 38,939,844  (86.7) | 22,001,337  (69.1) | 28,750,991  (70.6) | 18,876,514  (45.7) | 23,802,397  (63.3) | 10,094,529  (30.1) | 19,956,858  (55.4) | 4,352,361  (17.6) | 3,524,572  (20.8) | 9,000,981  (60.4) | 179,300,384  (55.6) |
| 0.75-1.50 | 4,103,634  (9.1) | 8,450,911  (26.6) | 9,731,324  (23.9) | 21,058,933  (51.0) | 9,814,950  (26.1) | 20,889,632  (62.3) | 12,257,592  (34.0) | 17,472,571  (70.5) | 8,866,347  (52.3) | 3,807,876  (25.6) | 116,453,770  (36.1) |
| >1.50 | 691,979  (1.5) | 951,129  (3.0) | 2,000,363  (4.9) | 1,205,973  (2.9) | 3,862,793  (10.3) | 2,487,624  (7.4) | 3,798,944  (10.5) | 2,941,364  (11.9) | 4,560,425  (26.9) | 2,091,714  (14.0) | 24,592,308  (7.6) |
| Total  (100) | 44,927,095  (100.0) | 31,821,324  (100.0) | 40,695,515  (100.0) | 41,286,963  (100.0) | 37,622,335  (100.0) | 33,522,305  (100.0) | 36,044,885  (100.0) | 24,766,296  (100.0) | 16,951,344  (100.0) | 14,900,571  (100.0) | 322,538,633  (100.0) |
|  | Sensitivity sub-index (decile) | | | | | | | | | | |
|  | 1st | 2nd | 3rd | 4th | 5th | 6th | 7th | 8th | 9th | 10th | Total (100) |
| 0.00-0.15 | 382,997  (0.4) | 504,394  (0.9) | 149,917  (0.5) | 400,742  (1.3) | 136,098  (0.5) | 102,739  (0.4) | 291,078  (1.1) | 57,550  (0.3) | 67,356  (0.4) | 99,300  (0.8) | 2,192,171  (0.7) |
| 0.15-0.75 | 55,831,439  (64.8) | 36,065,917  (67.7) | 18,592,265  (63.7) | 19,229,532  (60.0) | 14,255,205  (57.6) | 11,224,688  (48.0) | 11,973,076  (46.1) | 7,223,804  (41.9) | 3,794,055  (20.6) | 1,110,403  (9.2) | 179,300,384  (55.6) |
| 0.75-1.50 | 27,187,065  (31.5) | 13,306,006  (25.0) | 9,072,633  (31.1) | 11,228,355  (35.1) | 8,587,484  (34.7) | 9,650,569  (41.3) | 10,735,457  (41.3) | 6,921,855  (40.1) | 12,534,011  (68.0) | 7,230,335  (60.2) | 116,453,770  (36.1) |
| >1.50 | 2,788,662  (3.2) | 3,407,757  (6.4) | 1,383,488  (4.7) | 1,175,633  (3.7) | 1,779,716  (7.2) | 2,398,440  (10.3) | 2,987,703  (11.5) | 3,050,823  (17.7) | 2,044,087  (11.1) | 3,575,999  (29.8) | 24,592,308  (7.6) |
| Total  (100) | 86,190,163  (100.0) | 53,284,074  (100.0) | 29,198,303  (100.0) | 32,034,262  (100.0) | 24,758,503  (100.0) | 23,376,436  (100.0) | 25,987,314  (100.0) | 17,254,032  (100.0) | 18,439,509  (100.0) | 12,016,037  (100.0) | 322,538,633  (100.0) |
|  | Community health vulnerability index (decile) | | | | | | | | | | |
|  | 1st | 2nd | 3rd | 4th | 5th | 6th | 7th | 8th | 9th | 10th | Total (100) |
| 0.00-0.15 | 2,192,171  (3.0) | 0  (0.0) | 0  (0.0) | 0  (0.0) | 0  (0.0) | 0  (0.0) | 0  (0.0) | 0  (0.0) | 0  (0.0) | 0  (0.0) | 2,192,171  (0.7) |
| 0.15-0.75 | 65,466,734  (89.3) | 34,614,722  (92.3) | 28,499,057  (69.4) | 26,029,670  (80.6) | 17,271,512  (59.4) | 5,640,597  (18.8) | 1,681,994  (5.4) | 71,568  (0.3) | 10,195  (0.1) | 14,335  (0.1) | 179,300,384  (55.6) |
| 0.75-1.50 | 5,617,894  (7.7) | 2,882,225  (7.7) | 12,562,250  (30.6) | 5,772,576  (17.9) | 11,434,489  (39.3) | 22,851,643  (76.3) | 24,998,938  (79.9) | 17,349,969  (82.8) | 10,110,582  (64.6) | 2,873,204  (25.0) | 116,453,770  (36.1) |
| >1.50 | 0  (0.0) | 0  (0.0) | 0  (0.0) | 502,821  (1.6) | 358,055  (1.2) | 1,451,971  (4.8) | 4,623,511  (14.8) | 3,526,622  (16.8) | 5,527,023  (35.3) | 8,602,305  (74.9) | 24,592,308  (7.6) |
| Total  (100) | 73,276,799  (100.0) | 37,496,947  (100.0) | 41,061,307  (100.0) | 32,305,067  (100.0) | 29,064,056  (100.0) | 29,944,211  (100.0) | 31,304,443  (100.0) | 20,948,159  (100.0) | 15,647,800  (100.0) | 11,489,844  (100.0) | 322,538,633  (100.0) |


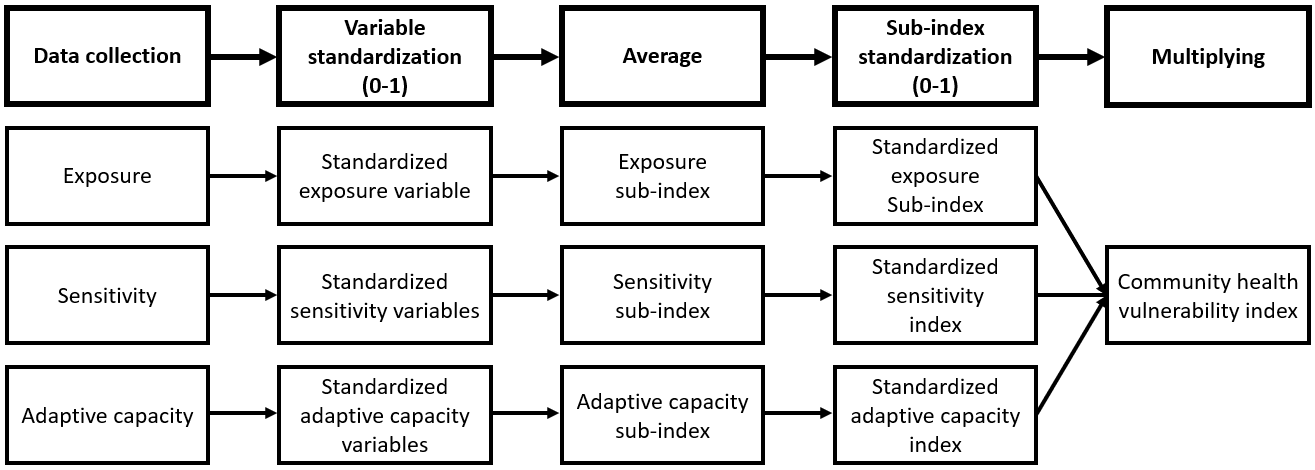


Supplementary Figure S1. Schematic figure of developing community health vulnerability index.


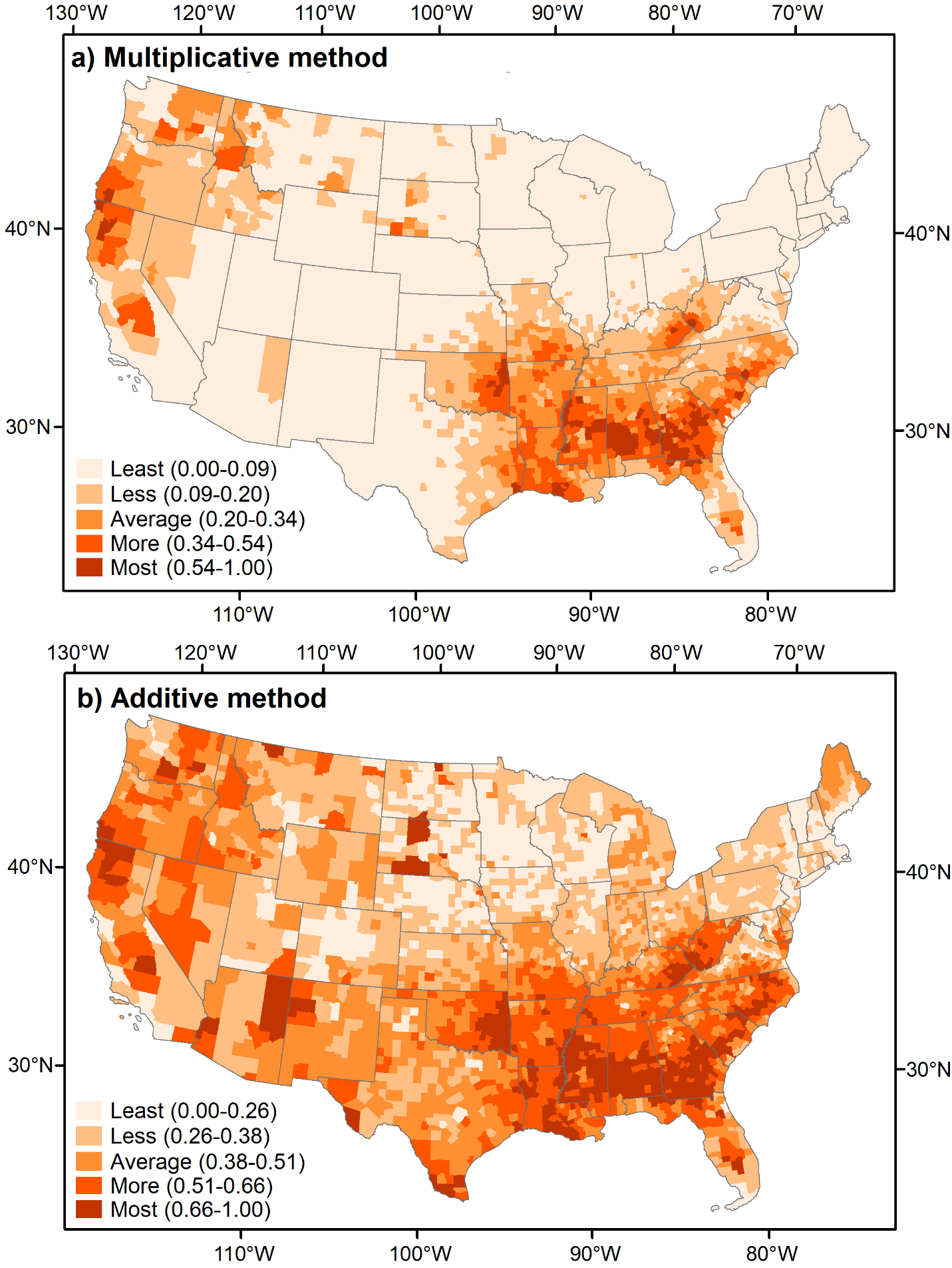


Supplementary Figure S2. CHVIs based on multiplicative method (a) and additive method (b)


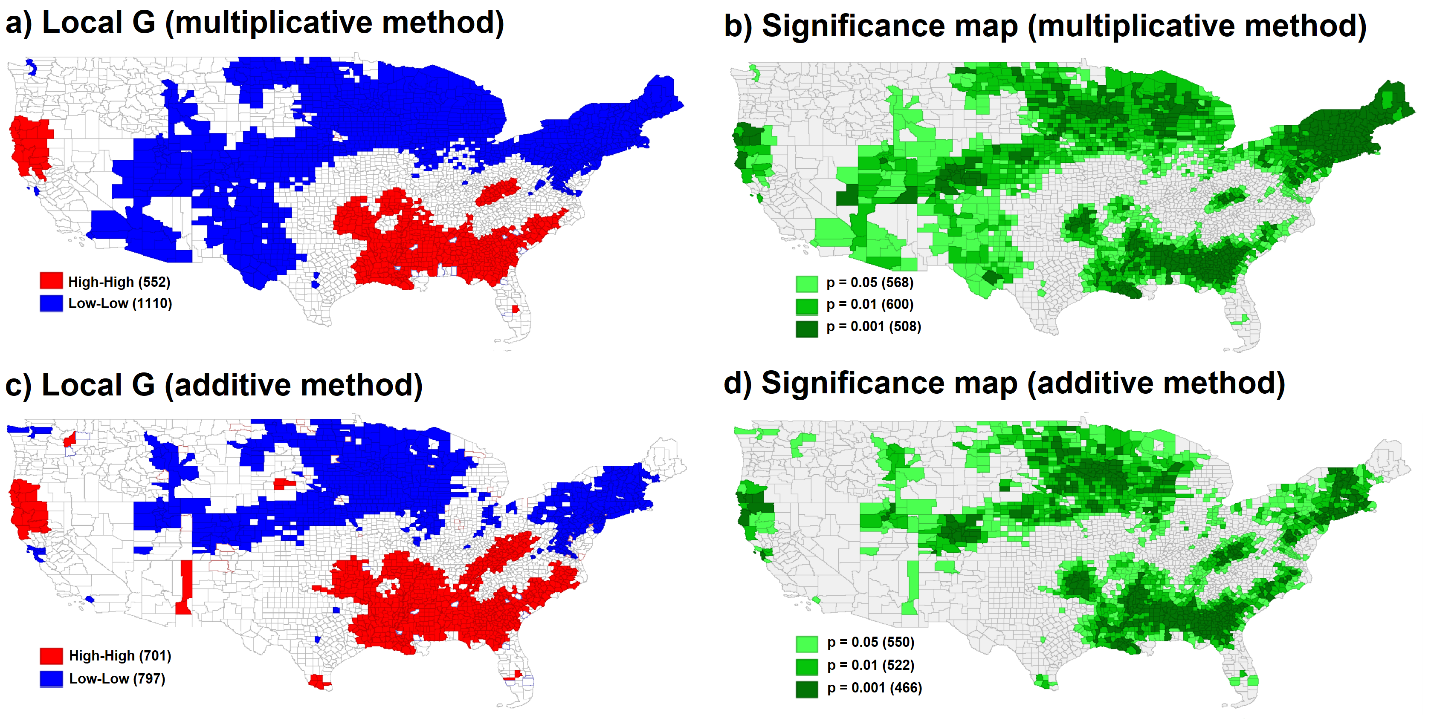


Supplementary Figure S3. Hot and cold spot analysis. We conducted ad-hoc analysis to find if there are any large differences between two spatial patterns of vulnerability indices. In this analysis, we used the Getis-Ord statistics to find hot spots and cold spots. Red counties represent the counties with high vulnerability and blue counties indicate the counties with low vulnerability.


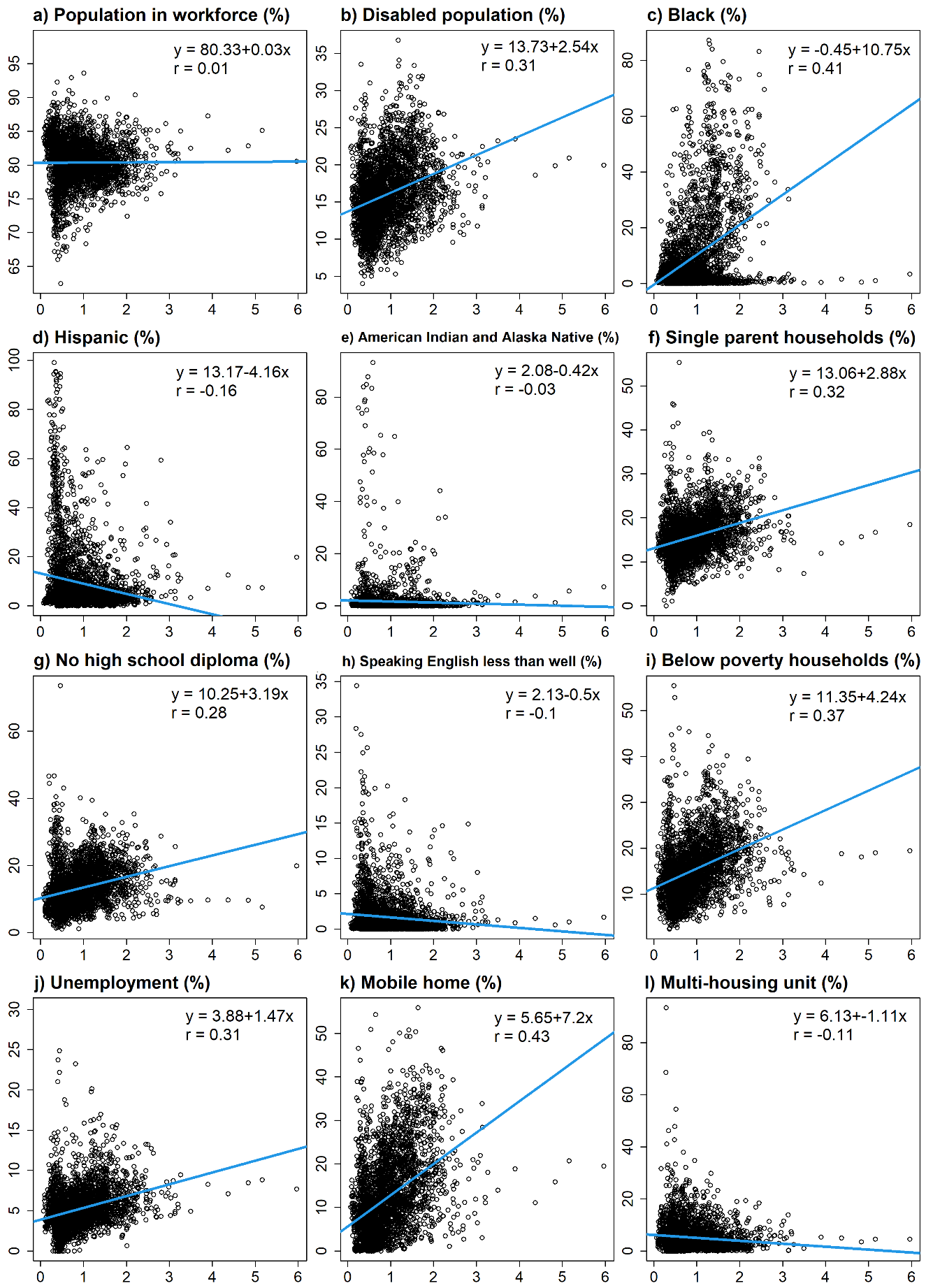


Supplementary Figure S4. Scatter plot of exposure and each adaptive capacity variable (continued).


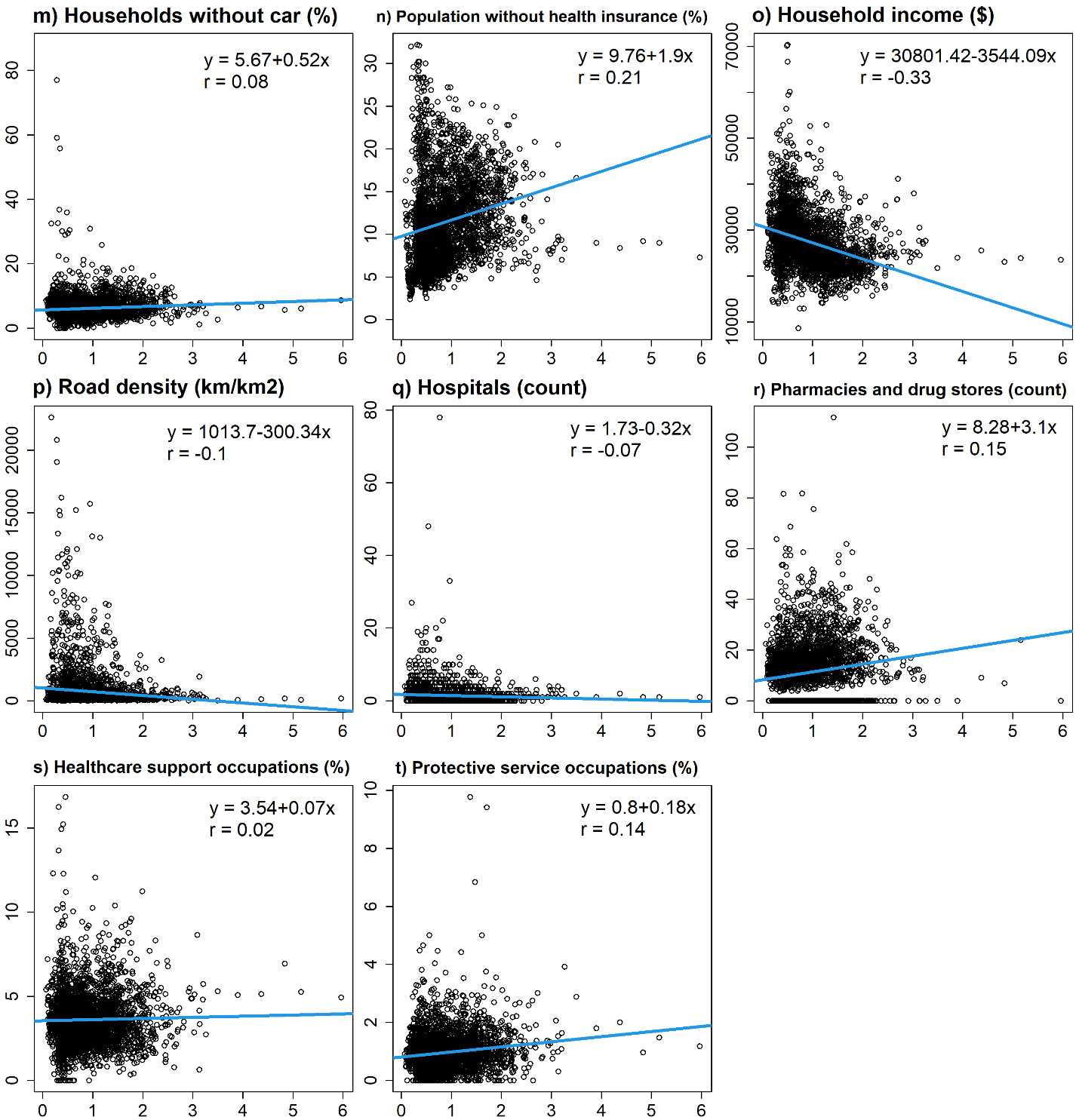


Supplementary Figure S4. Scatter plot of exposure and each adaptive capacity variable.


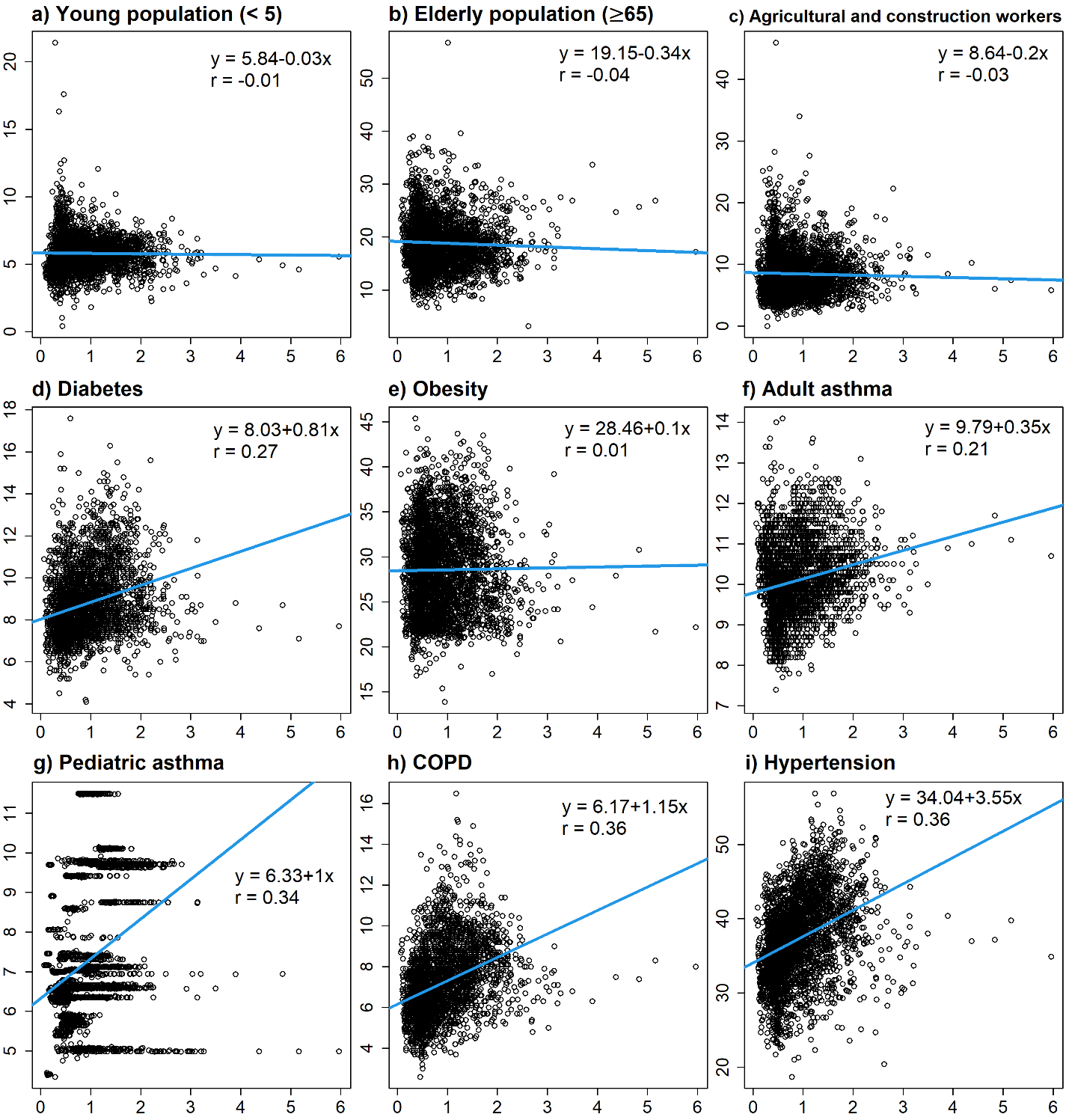
Supplementary Figure S5. Scatter plot of exposure and each sensitivity variable.


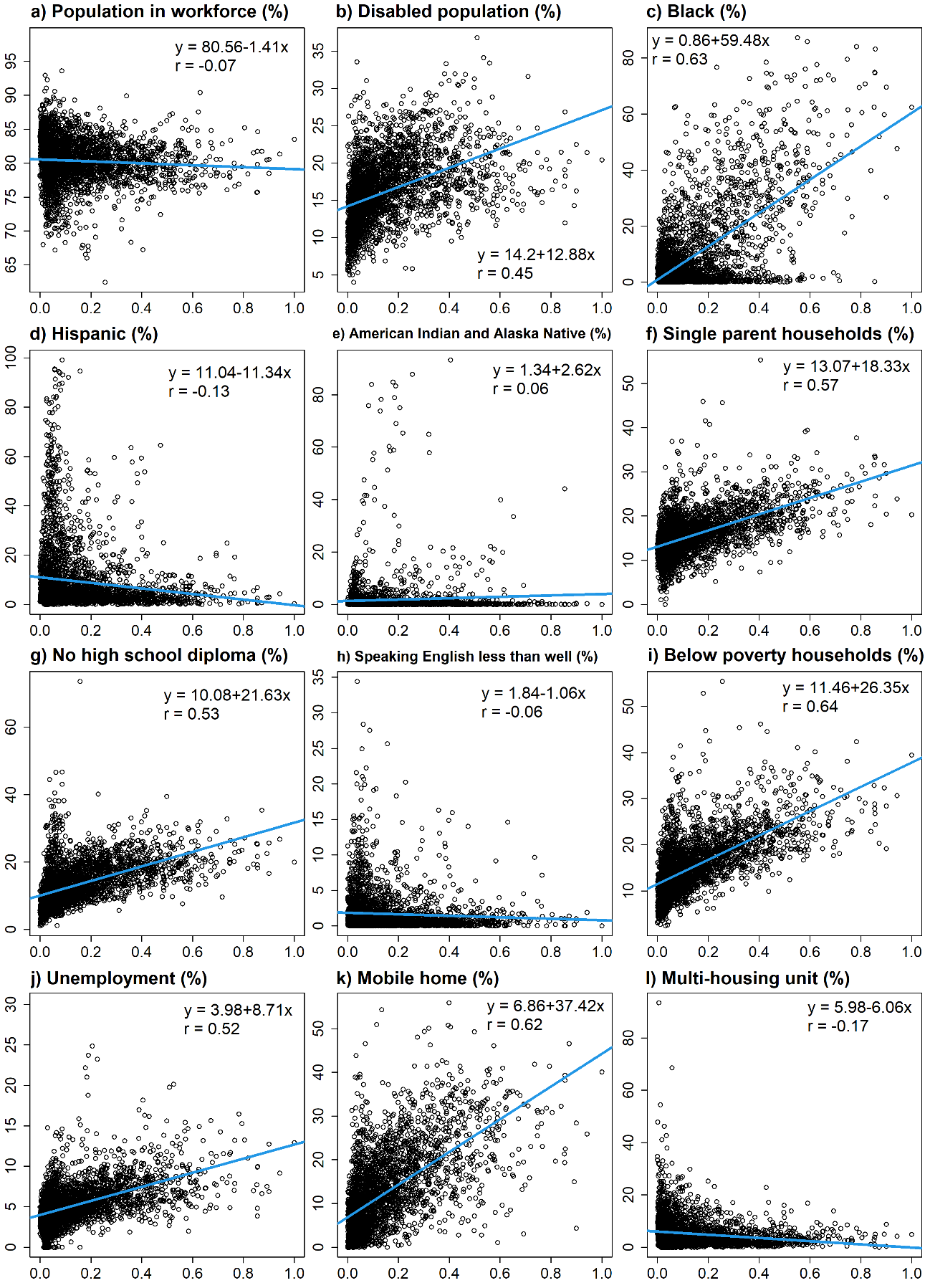


Supplementary Figure S6. Scatter plot of vulnerability index and each adaptive capacity variable (continued).


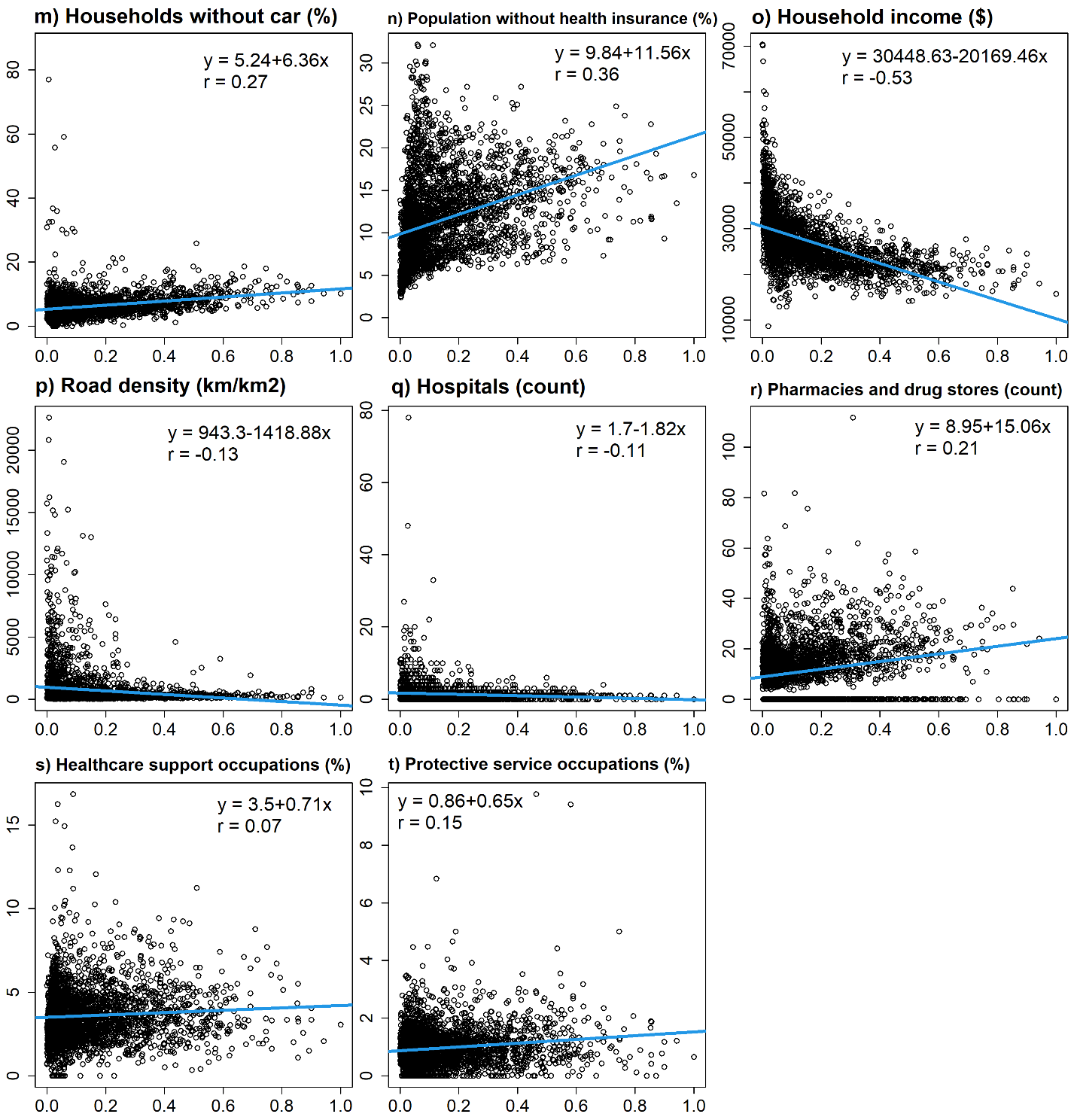


Supplementary Figure S6. Scatter plot of vulnerability index and each adaptive capacity variable.


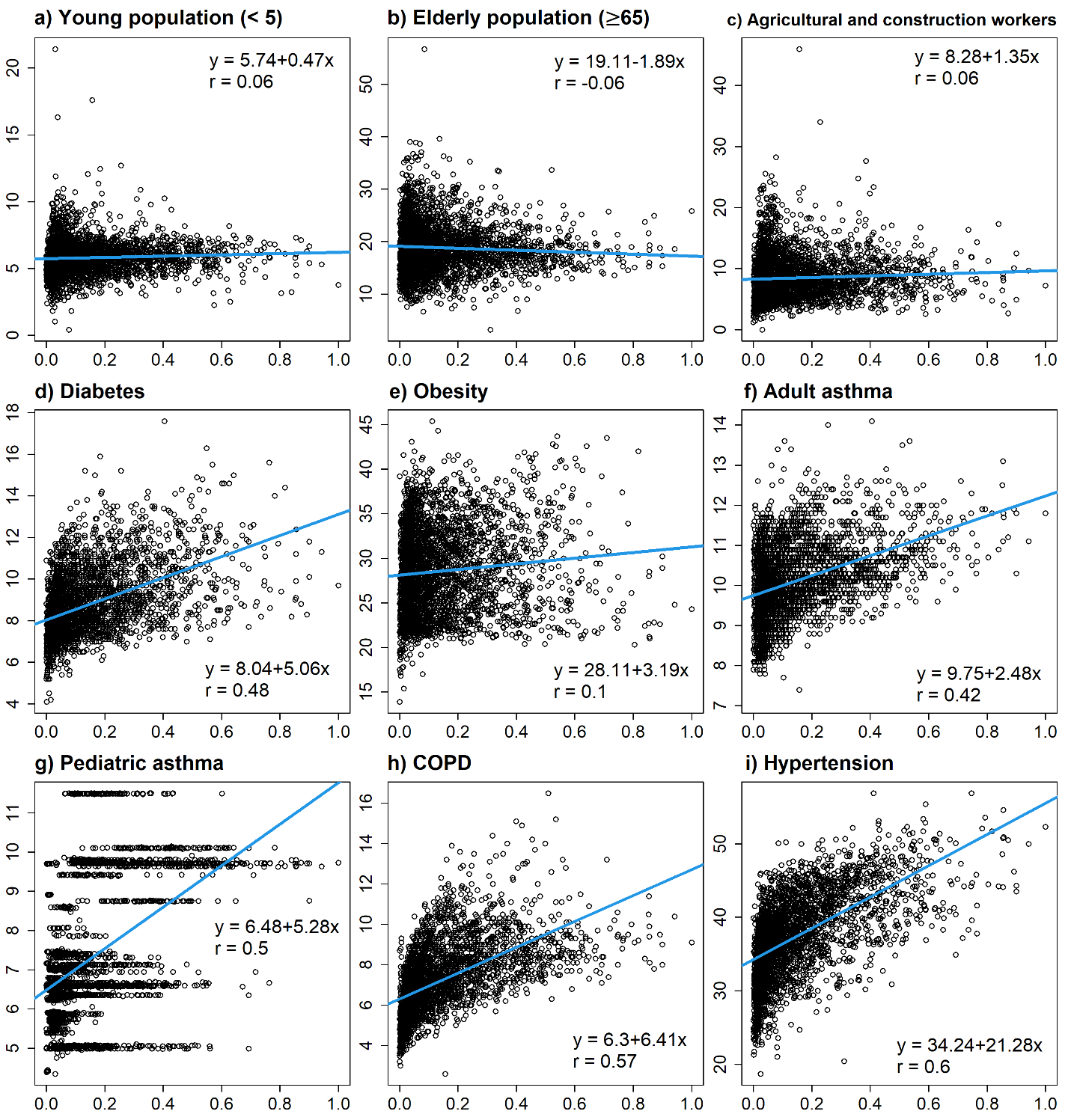


Supplementary Figure S7. Scatter plot of vulnerability index and each sensitivity variable.


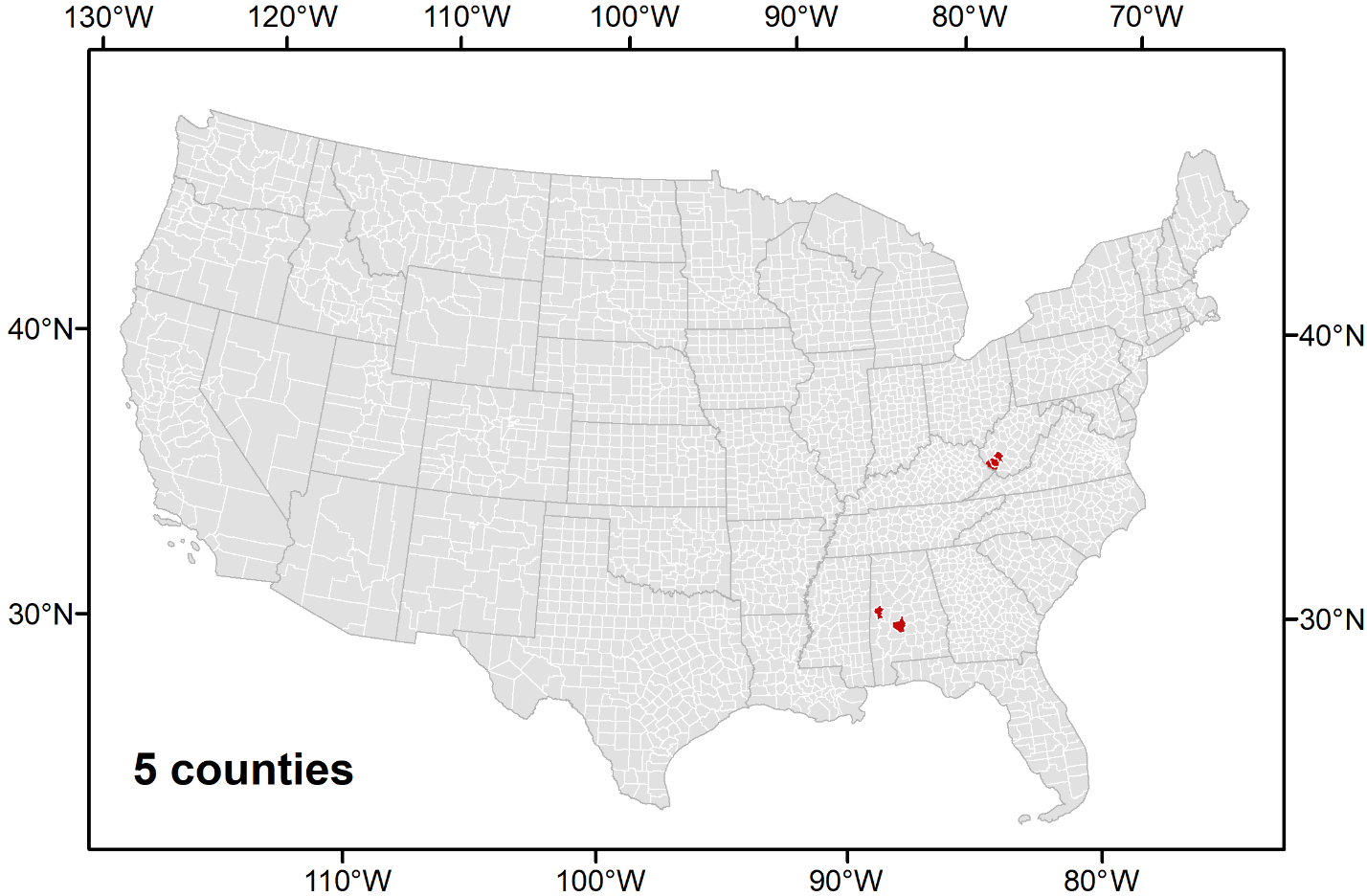


Supplementary Figure S8. Counties with the highest daily fire-PM_2.5_ exposure >1.50μg/m^3^, the highest adaptive capacity sub-index (>99th percentile), and the highest sensitivity sub-index (>99th percentile).
